# Transcriptome-Based WGCNA Reveals Hub Genes Involved in Copper Resistance of *Penicillium janthinellum* GXCR

**DOI:** 10.3390/ijms27073290

**Published:** 2026-04-04

**Authors:** Qin Zhang, Shaoke Huang, Abrar Khan, Haiman Gan, Jinzi Wang, Yongqiang Liu, Tianlin Teng, Feiyan Wei, Jian Xu, Xiaoling Chen

**Affiliations:** 1 Guangxi Key Laboratory for Polysaccharide Materials and Modifications, Guangxi Marine Microbial Resources Industrialization Engineering Technology Research Center, School of Marine Sciences and Biotechnology, Guangxi Minzu University, 158 University Road, Nanning 530008, China; qzhang1220@126.com (Q.Z.); 18378191309@163.com (S.H.); ghmas11@163.com (H.G.); wangjinzi@gxmzu.edu.cn (J.W.); liuyongqiang110@126.com (Y.L.); 18777167279@163.com (T.T.); wfy667066@163.com (F.W.); 2 National Key Laboratory of Non-Food Biomass Energy Technology, Guangxi Academy of Sciences, Nanning 530007, China; 3 State Key Laboratory for Conservation and Utilization of Subtropical Agro-Bioresources, College of Life Science and Technology, Guangxi University, 100 Daxue Road, Nanning 530004, China; abrargxu@gmail.com

**Keywords:** *Penicillium janthinellum*, heavy metal stress, transcriptome sequencing, WGCNA, hub genes

## Abstract

Filamentous fungi exhibit high heavy metal resistance; elucidating their resistance mechanisms is of practical importance for fungal utilization and for engineering other microorganisms. However, the molecular basis of copper tolerance in filamentous fungi remains poorly understood, with few studies addressing this specific trait. Previously, we isolated a copper-hyper-resistant strain, *P. janthinellum* GXCR, and generated two mutagenized derivatives, EC-6 and UC-8. To investigate copper resistance, wild-type GXCR (WT) and mutants EC-6 and UC-8 were subjected to integrated physiological, biochemical, and transcriptomic analyses. Copper tolerance followed the rank order: WT > UC-8 > EC-6. Supplementation with Mn^2+^ or exogenous proline enhanced copper resistance. Under copper stress, intracellular reactive oxygen species (ROS) levels increased in all strains, correlating dynamically with activities of superoxide dismutase (SOD), peroxidase (POD), and catalase (CAT), as well as malondialdehyde (MDA) content, with all exhibiting a biphasic response: an initial rise followed by a decline with increasing Cu^2+^ concentration. WT accumulated less Cu and Cd but more Cr (at high concentration) than the mutants. In contrast, intracellular Pb accumulation in all three strains decreased monotonically with rising Pb doses. RNA-seq of WT and EC-6 grown in TYB with 0, 0.5 and 3 mM Cu^2+^ identified 8 copper-resistance-related genes, verified by real-time quantitative reverse transcription PCR (RT-qPCR). Weighted gene co-expression network analysis (WGCNA) clustered genes into 10 modules; integrating physiological data identified 10 traits, and the four most correlated modules yielded 116 hub genes mostly linked to energy metabolism, cell components and transporters. *copA* and *ATP7*, encoding Cu^2+^-exporting ATPases, were identified as central regulators of copper homeostasis and key contributors to enhance copper tolerance. These findings provide molecular insights into copper resistance of filamentous fungi and valuable genetic targets for rational strain engineering.

## 1. Introduction

Heavy metals cannot be degraded in natural environments; while damaging soil, air and water, they also harm plants, animals and microorganisms, enter the human body through the food chain, and endanger health [1]. Heavy metals are defined as metals with a relative density greater than 5 g cm^−3^; they occur in dissolved or particulate forms, predominantly the latter, and are embedded in various minerals as essential constituents of the Earth’s crust [2]. Copper is a common and widely used heavy metal in industry, agriculture and daily life, and pollution generated during its processing and smelting is widespread [3]. Excess copper is toxic to organisms, and free copper ions within cells play a key role in the generation of reactive oxygen species [4]; therefore, copper was selected as the target element in this study. Microbial resistance to heavy metals varies greatly: fungi exhibit the highest resistance, followed by bacteria, whereas actinomycetes are the least resistant [5]. Therefore, elucidating copper resistance mechanisms in filamentous fungi is crucial not only for their genetic engineering, control, and utilization, but also for informing the modification of other strains and molecular drug design.

Genetically engineered strains often perform well under laboratory conditions, yet their efficacy in practical applications remains disappointing, the underlying reason is that the recipient organisms exhibit poor adaptation to complex environments, partly because of low resistance to heavy metals [6]—a factor that has received little attention. Therefore, it is necessary to use highly heavy-metal-resistant strains as starting points for genetic engineering or to investigate the resistance mechanisms of strains exhibiting heavy metal tolerance. Wei et al. [7] reported that *P. janthinellum* exhibits high copper resistance. The copper-transporting ATPase of GXCR strain was identified as a P_1B_-type ATPase, and 40 mM Cu^2+^ markedly induced expression of this gene, indicating that this transporter contributes to copper resistance—yet the core genes and mechanisms responsible for the exceptional metal resistance of GXCR remain unknown. P-type ATPases are a family of ion transporters that use ATP hydrolysis to drive the translocation of substrates across membranes; P_1B_-type ATPases specifically transport heavy metals such as copper [8,9].

Research has indicated that fungal resistance to heavy metals (e.g., copper) primarily relies on transcriptional regulation mechanisms: when intracellular copper concentrations increase, specific transcription factors rapidly activate or inhibit the expression of downstream target genes, thereby swiftly initiating resistance pathways such as metal chelation, efflux, or sequestration. This regulatory level plays a central role in fungal responses to copper stress [10].

In fungal copper-resistance assays, manganese serves as the cofactor of manganese superoxide dismutase (Mn-SOD) and enhances tolerance to copper-induced oxidative stress. Copper ions trigger intracellular reactive oxygen species (ROS) accumulation, whereas manganese alleviates copper-mediated oxidative damage by reinforcing antioxidant enzyme activities [11]. Within a certain range, organisms counteract dehydration by osmotic adjustment: cells actively accumulate solutes to lower osmotic potential, maintaining intracellular–extracellular pressure balance and preserving the integrity and functionality of membranes and walls. During this process, proline rapidly accumulates to levels several- to a hundred-fold higher than under normal conditions. Proline functions to (i) maintain osmotic balance between the cytoplasm and the environment, (ii) protect macromolecular conformation and stabilize proteins via its dipolar properties, and (iii) form hydrated colloids with intracellular compounds, providing osmoprotection [12,13].

Genetic manipulation of GXCR is challenging because conventional gene function analyses such as gene knockout or overexpression cannot be employed. The high-throughput fungal transformation systems currently used in most laboratories rely on Agrobacterium-mediated T-DNA insertional mutagenesis with selectable markers such as hygromycin [14], benomyl [15] or the herbicide Basta [16]; these markers are incompatible with large-scale mutant library construction in GXCR. As the wild-type GXCR strain exhibits extremely high intrinsic resistance to these antibiotics, we previously subjected the WT *P. janthinellum* strain GXCR to combined chemical–physical mutagenesis and obtained mutants with reduced heavy metal resistance.

This study aimed to determine the activities of antioxidant enzymes and the contents of non-enzymatic antioxidants in GXCR and two mutants, EC-6 and UC-8, under copper stress, as well as their intracellular accumulation of different heavy metals. The effects of manganese ions and exogenous proline supplementation on copper resistance were investigated, and performed transcriptome sequencing to examine gene expression differences under various stresses. We found that the wild-type and mutant strains exhibited distinct copper resistance on PDA and TYA media, and both proline and ATP enhanced copper tolerance. Through transcriptomic analysis and qPCR validation, we identified 38 transcripts and 14 copper-resistance-related genes. By integrating WGCNA with transcriptomic and physiological–biochemical data produced a visualized network and yielded 116 hub genes. These results provide theoretical foundations and data references for further investigation of copper and other heavy metal resistance mechanisms in *P. janthinellum*, for engineering application of GXCR, and construction of other highly resistant strains, offering both theoretical significance and practical value for remediating environmental heavy metal contamination.

## 2. Results

### 2.1. The Wild-Type Copper Tolerance Exceeds That of Its Mutants

Determining the tolerance of the three strains to heavy metal stress clarifies the biological characteristics of the organism; hence, the copper tolerance of each strain was measured on media containing graded copper concentrations.

Results showed that the WT strain exhibited significantly higher copper tolerance than both mutants in the two tested media. Specifically, copper concentrations required to inhibit growth reached 1000 mM on PDA and 800 mM on TYA for the WT. Across all strains, copper tolerance on PDA and PDB were greater than those on TYA and TYB. Among the two mutants, UC-8 displayed higher copper resistance (Table 1).

### 2.2. Copper Inhibits Mutant Growth More than the Wild-Type, While Manganese or Proline Enhances Copper Tolerance

**Figure 1:**
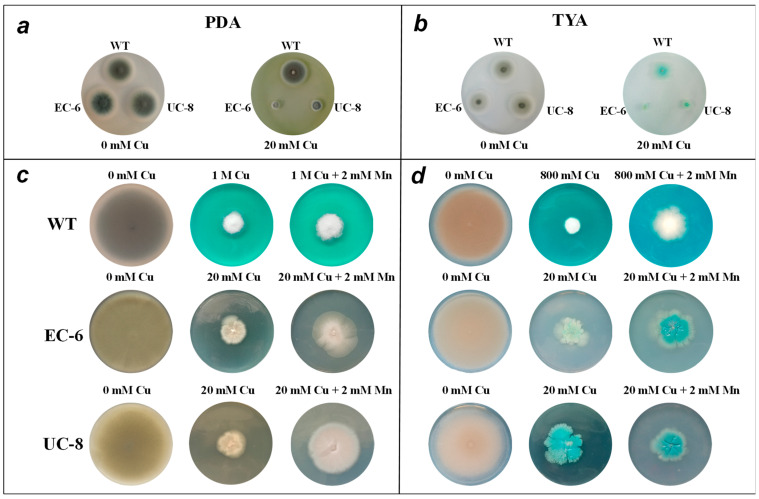
The colonial morphology of PDA and TYA. Condition of culture: (**a**): PDA, 32 °C, 4 d; (**b**): TYA, 32 °C, 4 d; (**c**): PDA, 32 °C, 10 d; (**d**): TYA, 32 °C, 10 d. Standard 9 cm circular Petri dishes.

As shown in Figure 1a, after 4 days of cultivation on copper-free PDA medium, the colonies of both mutant strains were slightly larger than those of the WT, and their spore production was also higher. Under 20 mM copper ion stress, the WT colonies were slightly reduced in size, while both mutant strains exhibited a significant decrease in colony size—with mutant EC-6 showing the smallest colonies (Figure 1a). This indicates that 20 mM copper ions markedly inhibited the growth of the mutant strains, whereas the WT remained largely unaffected, suggesting the WT possesses significantly higher copper tolerance than the mutants. Under 20 mM copper stress, spore production in the mutant strains decreased markedly compared to that under non-stressed conditions, while the WT exhibited minimal changes in spore production (Figure 1a). This further confirms that the mutants suffered greater inhibition of growth and reproduction than the WT. As shown in Figure 1b, all three strains developed distinct blue-pigmented hyphae on TYA medium supplemented with copper, confirming the precipitation of copper ions on the hyphal surfaces. Additionally, the WT showed less growth inhibition on PDA medium (Figure 1a) than on TYA medium (Figure 1b), suggesting that PDA is more conducive to enhancing the WT’s copper resistance compared to TYA.

Given the minimal impact of 20 mM copper ions on the WT, higher Cu^2+^ concentrations (800 mM in TYA medium and 1 M in PDA medium) were used to culture the WT, aiming to better investigate the effects of Cu^2+^ on the WT. As shown in Figure 1c, after 10 days of cultivation on stress-free PDA medium, all strains exhibited robust growth with substantial spore production. The spores appeared bluish-green, and mutant EC-6 produced more spores than the WT and mutant UC-8. Under copper stress, the colonies of all three strains shrank dramatically. The WT strain exposed to 1 M copper ions showed slightly smaller colonies than the mutant strains under 20 mM copper stress, indicating the WT’s superior stress tolerance compared to the mutants—consistent with the findings from cultivation under 20 mM copper stress (Figure 1a,b). The mutant strains exhibited an irregular circular morphology, while the WT showed no significant morphological changes. The WT demonstrated markedly superior tolerance to copper ions compared to the mutant strains, which may be attributed to milder disruption of cell wall synthesis, polar growth regulation, and oxidative stress-related signaling pathways by copper ions in the WT, or more effective compensatory mechanisms in the WT [17,18]. Spore production was extremely low in the WT under 1 M copper stress and in the mutant strains under 20 mM copper stress. The mutant strains produced more spores than the WT, and both strains exhibited white mycelia. Compared to spore production in all three strains under non-copper stress conditions, spore formation was markedly reduced (Figure 1c). After adding manganese ions to the copper-containing medium, the colony morphology of all three strains enlarged significantly, and spore production increased to some extent (Figure 1c,d), indicating that manganese ion supplementation promotes the growth of these strains under copper stress.

However, no growth enhancement was observed for UC-8 in TYA medium (Figure 1d). This discrepancy may arise from differences in copper-manganese interaction mechanisms: the WT and EC-6 likely counteract copper stress via Mn^2+^-activated Mn-SOD, Cu-Mn transporters, or antioxidant systems. In contrast, UC-8 exhibits defective copper efflux/chelation systems due to its mutation, and this protective mechanism may be suppressed under the chelating conditions of TYA medium [19].

Given that key genes in the proline metabolic pathway influence spore formation [20], we supplemented PDA and TYA media with different concentrations of proline and copper ions to investigate the effect of exogenous proline supplementation on the copper tolerance of the strains.

**Figure 2:**
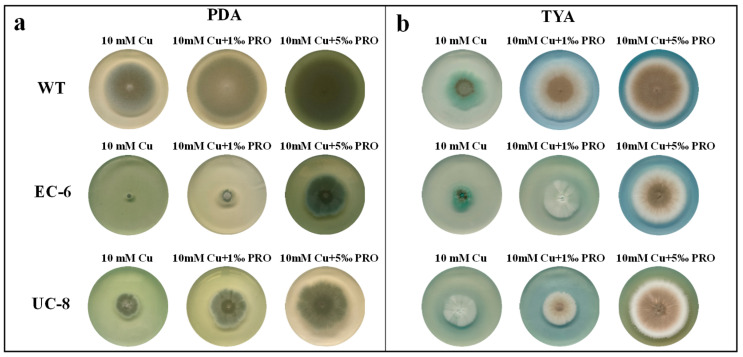
Colony morphology of the three strains grown on PDA and TYA supplemented with exogenous proline. Condition of culture: PDA, TYA, 10 d, 32 °C. Standard 9 cm circular Petri dishes. (**a**): PDA, 32 °C, 10 d; (**b**): TYA, 32 °C, 10 d.

On PDA medium, compared to the control group without proline supplementation, the addition of proline increased spore production and enlarged colony diameter in the strains. The spores exhibited a deeper cyan-green color, which is indicative of higher spore yield. As proline concentration increased, both colony diameter and spore production further increased. Under the same conditions, among the three strains, the WT produced the highest spore yield, followed by UC-8, while EC-6 produced the lowest spore yield (Figure 2a). Figure 2b, and f show that on TYA medium, compared to the non-proline-supplemented control, the addition of proline resulted in more regular colony morphologies, larger colony diameters, and increased spore production. Both colony diameter and spore production increased significantly with rising proline concentrations (Figure 2b). These results indicate that proline significantly promotes the growth of the strains. On proline-supplemented medium, all three strains exhibited increased spore production and larger colony diameters compared to those cultured on copper-containing medium without proline. The WT showed the highest spore production and largest colony diameter, followed by UC-8, while EC-6 exhibited the lowest spore production and smallest colony diameter.

### 2.3. WT Spore Germination Exceeds That of Mutants Under Copper or Chromium Stress, and Manganese Addition Improves Germination Rates

To investigate the effects of copper stress on spore germination in the three fungal strains, spore germination rates were determined under different concentrations of copper stress.

**Figure 3:**
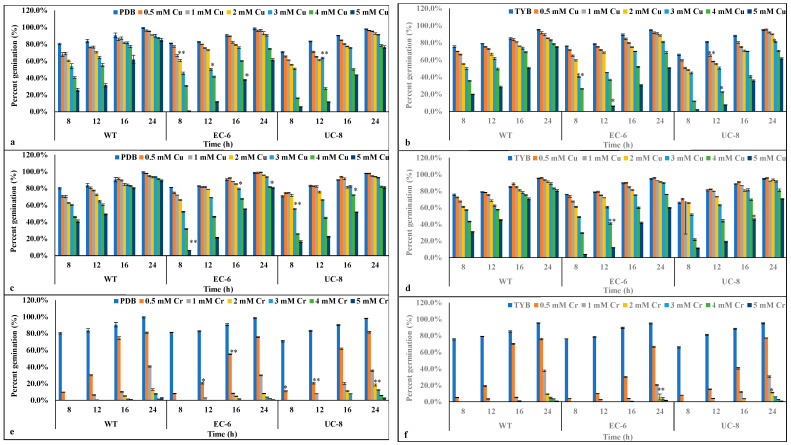
Effect of different heavy metal stress to percent germination of conidia. (**a**) PDB with Cu^2+^. (**b**) TYB with Cu^2+^. (**c**) PDB with Cu^2+^ + 2 mM Mn^2+^. (**d**) TYB with Cu^2+^ + 2 mM Mn^2+^. (**e**) PDB with Cr^6+^. (**f**) TYB with Cr^6+^. Significant differences (* *p* < 0.05; ** *p* < 0.01).

As shown in Figure 3a, the germination rates of the two mutants were significantly lower than that of the WT. In TYB liquid medium, the spore germination rate of the WT under 0.5 mM Cu^2+^ stress was lower than that in PDB medium at the same incubation time. Compared to spore germination rates in PDB, EC-6 spore germination was slower in TYB (Figure 3b). As shown in Figure 3c,d, adding 2 mM Mn^2+^ to copper-containing PDB or TYB liquid media increased the spore germination rates of all three strains at the same incubation time. This indicates that Mn^2+^ addition promoted spore germination, which is consistent with the results in Figure 2. As shown in Figure 3e, in PDB liquid medium containing Cr^6+^, the spore germination rates of all three strains were lower than those under equivalent copper stress concentrations. With increasing Cr^6+^ concentration, spore germination rates decreased significantly, and the highest rate did not reach 40%. At the same Cr^6+^ concentration, the spore germination rates of all three strains in TYB medium were lower than those in PDB medium (Figure 3f). The results in Figure 3 indicate that with increasing concentrations of copper and chromium ions, conidial germination time was delayed to varying degrees, and germination rates were significantly reduced. Under Cr^6+^ and Cu^2+^ stress conditions, the two mutants exhibited greater suppression of conidial germination than the WT. Overall, the spore germination rates of the three strains in PDB medium were slightly higher than those in TYB liquid medium.

### 2.4. Copper Stress Concentrations Affect ROS Levels and Antioxidant Enzyme Activities

ROS are one of the metabolic by-products in biological cells. They can regulate cellular physiological activities while also causing oxidative damage to organelles [21]. Under abiotic stress, the intracellular ROS balance is disrupted [22]. Since intracellular ROS levels serve as a crucial indicator for assessing biological stress responses, we investigated changes in ROS content and the activity of stress-tolerance-related enzymes in the microbial strains under stress conditions.

**Figure 4:**
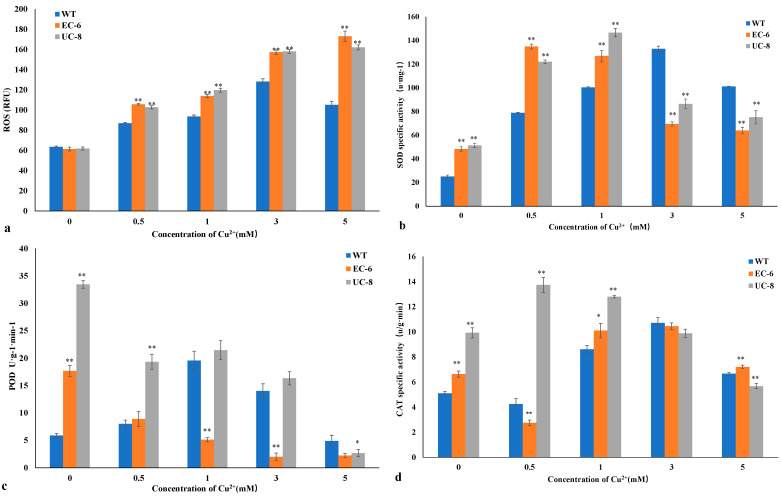
Effect of Cu^2+^ on the antioxidant enzyme activity of the three strains (**a**) The influence of Cu^2+^ on the content of reactive oxygen species in the three strains. (**b**) The influence of Cu^2+^ on the SOD activity of the three strains. (**c**) The influence of Cu^2+^ on the POD activity of the three strains. (**d**) The influence of Cu^2+^ on the CAT activity of the three strains. Note: Compare EC-6 and UC-8 with WT. Significant differences (* *p* < 0.05; ** *p* < 0.01).

The protein standard curve is shown in Figure S1. As shown in Figure 4a, the ROS content of the three strains gradually increased with rising Cu concentration, with EC-6 and UC-8 maintaining essentially the same ROS levels. The WT strain exhibits the lowest ROS content.

SOD activity is a crucial physiological indicator for assessing an organism’s response to abiotic stress. Under stress conditions, yeast SOD activity increases, which contributes to enhanced stress tolerance [23]. For all three strains, SOD activity exhibited an initial increase followed by a decrease within the 0–5 mM Cu^2+^ range. Specifically, SOD activity in the WT, EC-6, and UC-8 peaked at 3 mM, 0.5 mM, and 1 mM Cu^2+^, respectively (Figure 4b). The SOD activity levels followed the order: WT > UC-8 > EC-6, which is consistent with their respective copper tolerance levels. Subsequently, as copper concentration increased, the rate of superoxide anion radical accumulation exceeded the scavenging capacity of SOD, leading to a decrease in SOD activity. The differences in the copper concentrations at which SOD activity peaked among the three strains may underlie their varying copper tolerance levels.

POD plays a crucial role in cellular redox metabolism [22], functioning to scavenge reactive oxygen species and protect enzyme proteins. POD activity is an important physiological indicator for assessing organismal responses to abiotic stress [24]. As shown in Figure 4c, the trends in POD activity differed between the two mutants and the WT strain. WT POD activity increased with rising copper concentration, peaking at 1 mM Cu^2+^ before declining. In contrast, POD activity in EC-6 and UC-8 generally decreased progressively as copper concentration increased. When comparing EC-6 with the WT, approximately 35% of the differentially expressed transcripts (DETs) related to enzymatic antioxidants in EC-6 were downregulated under stress conditions, with POD-related DETs accounting for the majority (at least 89%). Thus, POD activity may be one factor contributing to the WT’s significantly higher copper tolerance compared to the mutants.

CAT is a ubiquitous enzymatic scavenger in organisms that acts synergistically with SOD and POD [22] to eliminate hydrogen peroxide generated by intracellular redox reactions. Its activity serves as a crucial physiological indicator of an organism’s stress tolerance. As shown in Figure 4d, the trends in CAT activity were similar for the WT and EC-6. In contrast, the CAT activity of mutant UC-8 peaked at 0.5 mM Cu^2+^ and gradually declined thereafter.

### 2.5. Heavy Metal Concentration Affects Intracellular Metal Accumulation

To investigate the tolerance mechanisms of wild-type and mutant strains of *P. janthinellum* under heavy metal stress, the strains were cultured in TYB medium supplemented with different heavy metal salts. Subsequently, the intracellular heavy metal content was determined using atomic absorption spectrophotometry.

**Figure 5:**
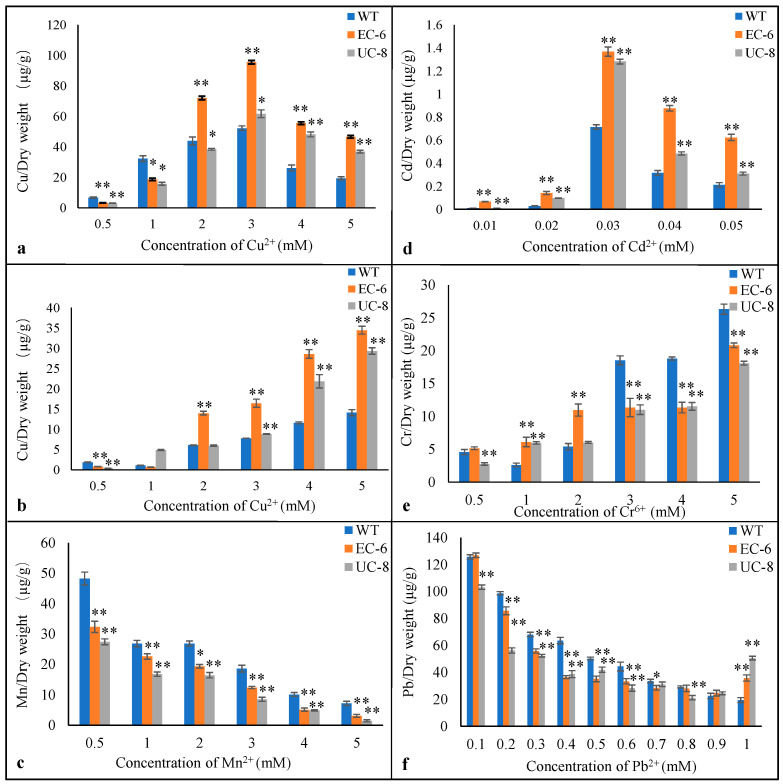
The content of heavy metal inside the cells. (**a**) TYB with Cu^2+^. (**b**) TYB with Cu^2+^ + 2 mM Mn^2+^. (**c**) TYB with 2 mM Mn^2+^. (**d**) TYB with Cd^2+^. (**e**) TYB with Cr^6+^. (**f**) TYB with Pb^2+^. Note: compare EC-6 and UC-8 with WT. Significant differences (* *p* < 0.05; ** *p* < 0.01).

In copper-containing TYB liquid medium, the intracellular copper content of all three strains increased significantly with rising extracellular copper concentration. At 0.5–1 mM Cu^2+^, the WT exhibited the highest intracellular copper content. At 3–5 mM Cu^2+^, the intracellular copper content followed the order: EC-6 > UC-8 > WT (Figure 5a). This suggests that all strains simultaneously sensed copper stress and initiated intracellular copper efflux responses. The higher copper tolerance of the WT compared to the mutant strains may be associated with its ability to maintain lower intracellular copper ion levels. When 2 mM Mn^2+^ was added to copper-containing TYB liquid medium, the intracellular copper levels of all three strains increased with rising copper concentration but remained significantly lower than those under Mn^2+^-free conditions (Figure 5b). This indicates that Mn^2+^ addition reduces intracellular copper ion content, likely by altering copper efflux mechanisms compared to Mn^2+^-free conditions [25]. The trend in intracellular manganese content differed from that of copper (Figure 5c), showing an overall decreasing pattern. In cadmium-containing TYB liquid medium, the intracellular cadmium levels of all three strains increased with rising cadmium concentrations, peaking at 0.03 mM Cd^2+^ before declining (Figure 5d). This pattern is consistent with the response to copper stress (Figure 5a), suggesting that these strains share common response and exclusion mechanisms for copper and cadmium stress. In chromium-containing TYB liquid medium (Figure 5e), the intracellular chromium levels in WT cells first decreased at 1 mM Cr^6+^ and then gradually increased. The intracellular chromium levels in EC-6 cells increased and stabilized at 2 mM Cr^6+^, while those in UC-8 cells increased with rising extracellular chromium concentrations. In lead-containing TYB liquid medium (Figure 5f), the intracellular lead levels of all three strains decreased with increasing extracellular lead, indicating that upon lead entry, the strains rapidly initiated lead efflux responses. Their lead efflux capacity exceeded that for other metals, effectively preventing lead from entering cells, and this efflux capacity was enhanced with higher extracellular lead concentrations.

### 2.6. Transcriptome Analysis and RT-qPCR Validation

In fungal cells, transcriptional regulatory mechanisms play a central role in responding to heavy metals such as copper. To investigate copper resistance mechanisms, two strains were selected for transcriptome analysis: the WT, which exhibits the highest copper resistance, and the EC-6 mutant, which exhibits the lowest copper resistance. WT and EC-6 were separately cultured in TYB medium containing 0, 0.5 mM, and 3 mM Cu^2+^. Total RNA was extracted from the cultured strains and subjected to transcriptome sequencing. The sequencing generated a total of 129 million reads with an average length of 93.60 bp, producing over 2 GB of data and achieving an effective read rate of 85.51%. Valid reads from all transcripts were pooled and assembled, yielding 47,407 transcripts with lengths exceeding 93.60 bp. After removing redundant sequences, 32,585 unigenes were obtained, among which 4424 unigenes had lengths exceeding 2000 bp.

Differentially expressed genes were defined based on the log2-transformed ratio of transcript counts for the same gene between two samples (e.g., A and B) cultured under identical conditions (A vs. B), representing the fold change in gene expression. A gene was considered up-regulated when the log2 value exceeded 1 and down-regulated when the log2 value was less than 1. Transcripts meeting these criteria were designated as differentially expressed transcripts (DETs). DETs identified from the comparison WT (Cu stress) vs. WT (control) represented those differentially expressed in the wild-type GXCR strain under Cu stress.

**Figure 6:**
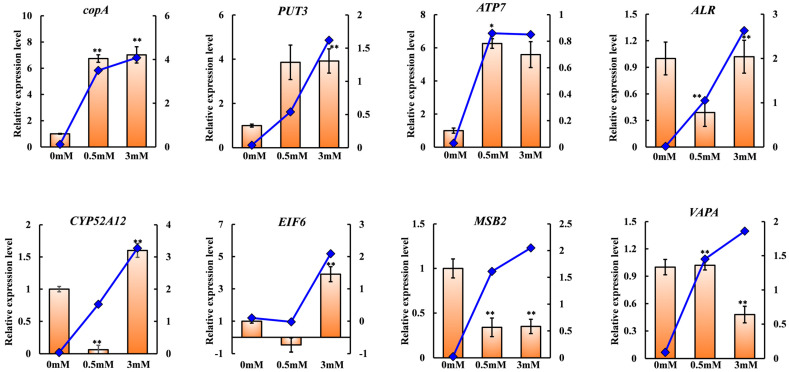
RT-qPCR validation of RNA-Seq data. The bar charts represent 2^^ΔΔCt^ values from RT-qPCR, while the line charts represent transcriptome gene expression levels (FPKM). * indicates that the differences in gene expression levels between 0 mM copper concentration and 0.5 mM and 3 mM copper concentrations are statistically significant at *p* < 0.05, while ** indicates that the differences in gene expression levels between 0 mM copper concentration and 0.5 mM and 3 mM copper concentrations are statistically highly significant at *p* < 0.01.

As the copper stress concentration increased, both up- and down-regulated differentially expressed transcripts in GXCR increased [17]. The qPCR results were then compared with the transcriptomic sequencing data (Figure 6; Tables S1–S6) and subjected to statistical analysis.

In this experiment, based on the two comparison groups “WT-TY3 VS WT-TY0” and “WT-TY0.5 VS WT-TY0”, a total of 8 copper-related genes were selected for RT-qPCR validation, and the results were largely consistent with the RNA-Seq data. Differences observed between the two datasets may stem from discrepancies in the detection principles of transcriptomic sequencing and RT-qPCR, leading to a certain degree of inconsistency [26,27]. The 8 genes are *copA*, *PUT3*, *ATP7*, *ALR*, *CYP52A12*, *EIF6*, *MSB2*, and *VAPA*, respectively. Core pathways closely associated with copper stress include: (1) copper efflux and homeostasis pathways (*copA*, *ATP7* genes); (2) proline metabolism pathways (proline dehydrogenase, *PUT3* gene); (3) mitochondrial electron transport chain (ATP synthase H chain); (4) cell wall integrity/stress response modules (*PSTPIP1*, *PPP2C* genes).

### 2.7. WGCNA Identified Key Gene Modules for Copper Resistance

WGCNA constructs gene co-expression networks (nodes = genes, edges = correlations) to predict gene functions and identify key phenotype-related genes [28,29]. To further explore the relationship between copper resistance and DEGs. In this study, WGCNA was employed to integrate transcriptomic sequencing data of *P. janthinellum* with physiological and biochemical results. Transcripts were classified into several co-expression modules, which were further associated with 10 traits. Correlation coefficients between the modules and traits were calculated to reveal the associations between gene modules and target traits.

The 10 traits are as follows: 1. Cu: Intracellular copper content in the microorganism; 2. Cu(Mn): Intracellular copper content under combined copper-manganese stress; 3. Mn(Cu): Intracellular manganese content under combined copper-manganese stress; 4. Biomass: Microbial biomass after 72 h of cultivation in copper-containing TYB medium; 5. ROS: ROS content after 72 h of copper stress; 6. SOD: SOD enzyme activity after 72 h of copper stress; 7. POD: Podophyllotoxin oxidase activity after 72 h of copper stress; 8. CAT: Catalase activity after 72 h of copper stress; 9. MDA: Malondialdehyde content after 72 h of copper stress; 10. Proline: Proline content after 72 h of copper stress.

**Figure 7:**
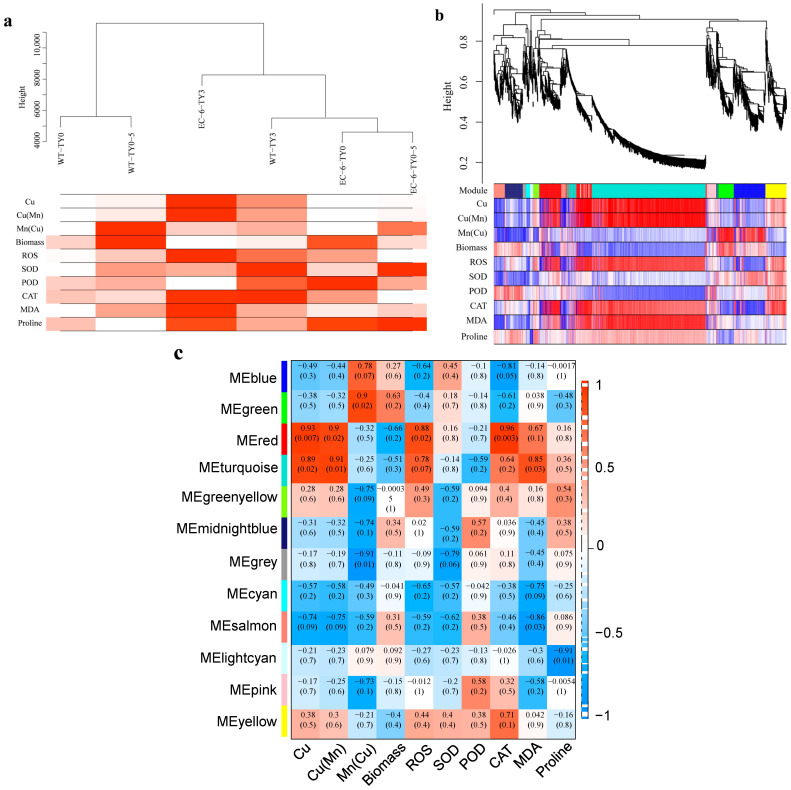
Key gene modules for copper tolerance screened by WGCNA. (**a**) Sample dendrogram and trait heatmap. “Height” is an internal distance of the clustering algorithm, unitless and not indicative of the actual measured values of ROS, Biomass, etc. The wild-type appears in different branches because the clustering object is the indicator rather than the genotype; different indicators find their “neighbors” based on their own expression patterns, independent of genotype. (**b**) Gene dendrogram with trait. Traits indicate the correlation between the genes in the sample and their respective modules. Red indicates a positive correlation, while blue indicates a negative correlation. (**c**) Module Trait Correlation. The correlation between traits and modules is indicated by numerical values. The closer the value is to 1, the higher the positive correlation between this trait and the module; the closer the value is to −1, the higher the negative correlation between this trait and the module. The numbers in parentheses are *p* values. The smaller the value, the higher the significance.

Figure 7a presents a hierarchical clustering dendrogram of samples based on gene expression levels, with a numerical heatmap of sample-related traits superimposed. The results show that WT-TY0 and WT-TY0.5 exhibit the highest correlation. The intensity of red indicates the module most strongly associated with each sample’s traits; deeper red signifies stronger association.

Genes were clustered into several modules, which were displayed in different colors. Combined with trait data, the correlations between genes in samples and their respective modules were indicated: red signifies a positive correlation, and blue denotes a negative correlation. Results showed that there were numerous modules highly correlated with both the Cu and Cu(Mn) traits, and these modules exhibited relatively high correlation coefficients, making them key targets for subsequent analysis (Figure 7b). All modules were divided into 12 categories, each represented by a different color (Figure 7c).

### 2.8. WGCNA-Based Identification of Copper-Resistance Hub Genes

**Figure 8:**
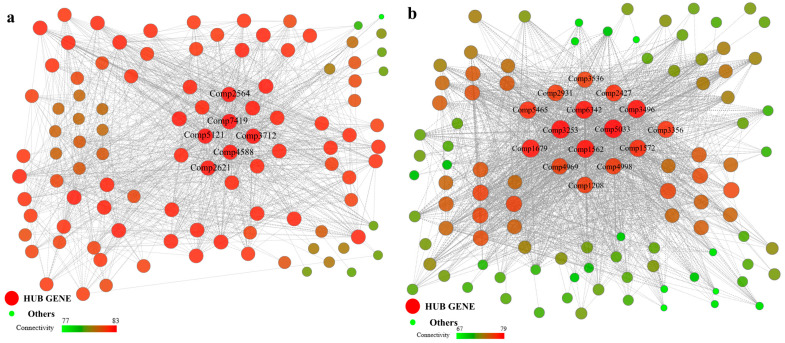
Screening of hub genes for copper resistance in *P. janthinellum* from the trait-specific module. (**a**) The correlation network of MEblue module. (**b**) The correlation network of MEred module.

To visually analyze gene correlations and identify hub genes, a gene co-expression network diagram for the 12 modules was constructed using Cytoscape software, version 3.6.1. The MEblue module showed a strong positive correlation with the Mn(Cu) trait and a strong negative correlation with the CAT trait, yielding 6 hub genes (Figure 8a). The MEgreen module showed a very strong positive correlation with the Mn(Cu) trait, a strong positive correlation with the Biomass trait, and a negative correlation with the CAT trait; 21 hub genes were identified (Figure S2). The MEred module showed extremely strong positive correlations with the Cu trait, Cu(Mn) trait, ROS trait, and CAT trait, and also exhibited a relatively strong positive correlation with the MDA trait. This indicates that this module is highly correlated with copper resistance in the strain and is one of the more important modules among the 12; a total of 15 hub genes were screened (Figure 8b). The MEturquoise module, similar to MEred, showed extremely strong positive correlations with the Cu trait, Cu(Mn) trait, ROS trait, and CAT trait, though its correlation coefficients were slightly lower than those of MEred. However, it exhibited the strongest positive correlation with the MDA trait among all modules, yielding 12 hub genes (Figure S3). The MEgreenyellow module showed a relatively strong negative correlation with the Mn(Cu) trait and a relatively strong positive correlation with the Proline trait, yielding 9 hub genes (Figure S4). The MEmidnightblue module exhibited a negative correlation with the Mn(Cu) trait, similar to the MEgreenyellow module; however, it differed in showing a relatively strong positive correlation with the POD trait, yielding 8 hub genes (Figure S5). The MEgrey module exhibited the strongest negative correlation with the Mn(Cu) trait and also showed a strong negative correlation with the SOD trait, yielding 6 hub genes (Figure S6). The MEcyan module displayed negative correlations with all 10 traits, with a particularly strong negative correlation with the MDA trait, yielding 8 hub genes (Figure S7). The MEsalmon module showed strong negative correlations with the Cu trait, Mn(Cu) trait, and MDA trait, yielding 8 hub genes (Figure S8). The MElightcyan module exhibited negative correlations with all traits except Mn(Cu) and Biomass, with the strongest negative correlation observed for Proline, resulting in 6 hub genes (Figure S9). The MEpink module showed a strong positive correlation with the POD trait and a strong negative correlation with the Mn(Cu) trait, yielding 8 hub genes (Figure S10). The MEyellow module exhibited a strong positive correlation with the CAT trait, yielding 9 hub genes (Figure S11).

Analysis of the gene co-expression network diagram for the 12 modules identified 116 hub genes (see Supplementary Figures S2–S11), along with their annotation information and differential fold changes across sample comparisons (see Supplementary Tables S7–S13).

## 3. Discussion

The copper tolerance of the strains varied significantly across different media containing varying heavy metal concentrations. Due to their relatively lower nutrient content, TYA and TYB media generally exhibited lower copper tolerance values for the strains in both solid and liquid forms (Figure 1c,d). Regarding heavy metal resistance, under 20 mM Cu^2+^ stress, the growth of the WT strain was almost unaffected, while the mutants EC-6 and UC-8 showed significant growth inhibition (Figure 1a,b). This indicates that the WT strain possesses high copper resistance, whereas the two mutants exhibit significantly lower copper tolerance than the WT strain. The resistance levels decreased in the order of WT > UC-8 > EC-6 (Figure S12). The addition of manganese ions promoted the growth of all three strains to varying degrees (Figure 1c,d). It is speculated that manganese ions in the medium provide multiple cofactors for strain growth [30] and enhance the activity of enzymes that utilize manganese ions as coenzymes [31]. The addition of proline to the medium enhanced the growth capacity of all three strains (Figure 2). This indicates that under copper stress, exogenous proline can reduce cellular osmotic pressure, alleviate the inhibitory effect of osmotic stress on strain growth, and mitigate the toxicity of copper ions to cells.

Analysis of the spore germination rates of the three strains in PDB and TYB media revealed that, due to the nutrient richness of PDB, the time required for spore germination to reach 80% was shorter than that in TYB medium. As the copper concentration increased, the spore germination time was delayed. Under identical conditions, the addition of 2 mM Mn^2+^ resulted in increased spore germination rates at the same time points (Figure 3c,d). This may occur because both manganese and copper ions are divalent cations that compete for copper ion transport channels in liquid media. Furthermore, manganese is an essential trace element for organismal growth [32], and its presence directly or indirectly enhances microbial heavy metal resistance. Under chromium stress, the spore germination rate of *P. janthinellum* decreased sharply (Figure 3e,f), indicating that chromium stress exerts greater toxicity than copper stress on this strain.

POD plays a crucial role in cellular metabolism [33], scavenging intracellular peroxides and protecting enzymes and proteins. Research indicates that copper ions counteract oxidative stress by influencing antioxidant enzyme activity. Intracellular excess copper (Cu) enhances the transcription of antioxidant enzymes and modifies/processes their protein structures. Furthermore, excessive copper ions induce massive ROS production, which causes damage to macromolecules such as nucleic acids, proteins, carbohydrates, and lipids. This ultimately disrupts the structure and expression system of antioxidant enzymes [34]. Under copper stress, *copA* and *ATP7* were significantly downregulated in the WT-TY3 vs. EC-6-TY3 group; this downregulation impaired copper efflux, leading to ROS accumulation and subsequent disruption of the antioxidant defense system. It is thus inferred that lower copper concentrations induce elevated SOD, POD, and CAT activities to counteract stress. Under high copper stress, however, the sharp increase in intracellular ROS reduces SOD, POD, and CAT activities, causing severe oxidative damage and potentially leading to cell death. This aligns with the results in Figure 4b–d.

The main pathway for fungi to adsorb heavy metal ions under heavy metal stress is that chitin and glucose in the cell wall bind to heavy metal ions through functional groups on the cell membrane and transport them into the cell [35]. The intracellular copper content of the three strains showed an initial increase followed by a decrease (Figure 5a). This trend is speculated to occur because under low copper stress concentrations, cells absorb copper ions and subsequently efflux them via active transport mechanisms. As copper concentrations increase, intracellular ROS levels rise. At this stage, the activity of multiple antioxidant enzymes and the content of antioxidants within the cells increase, leading to higher energy demands. This, in turn, reduces the rate of active transport, inhibiting copper absorption. Consequently, the efflux rate of copper ions increases, leading to a decrease in intracellular copper levels. Under co-stress with manganese ions, intracellular copper levels in all three strains decreased significantly under equivalent stress conditions. This likely occurs because manganese ions enhance intracellular antioxidant enzyme activity, accelerating ROS clearance. This alters cell membrane permeability, thereby inhibiting heavy metal ion entry into cells. Furthermore, under copper stress, the intracellular copper content of the WT strain was significantly lower than that of the two mutants, suggesting differing levels of stress resistance.

Spore production is associated with the *PUT3* gene, a transcription activator that activates key genes in the proline metabolic pathway, thereby regulating spore formation [20]. Proline dehydrogenase provides energy and reducing power to support spore formation [36]. *ATP7* and *copA* function as copper efflux ATPases; under copper stress, elevated ROS levels inhibit spore production, making copper homeostasis a prerequisite for sporulation [36,37,38]. Transcriptome studies reveal that differential expression of *PUT3*, proline dehydrogenase, *copA*, and *ATP7* contributes to the disparity in copper tolerance between the WT and EC-6 strains. In yeast and mammals, the *ATP7* protein undergoes retrograde transport from the Golgi apparatus to vesicles to the plasma membrane upon elevated copper concentrations, achieving copper efflux or compartmentalization through its altered subcellular localization [39]. When comparing EC-6 to WT under 0, 0.5, and 3 mM Cu stress, at least 82% of downregulated DETs in EC-6 were associated with *ATP7*, accounting for at least 56% of all downregulated DETs. Therefore, the high Cu accumulation in EC-6 [38] is likely due to failure of Cu efflux, resulting from impaired intracellular Cu translocation. *ATP7* remains trapped in the Golgi apparatus, unable to reach the plasma membrane, ultimately disrupting the efflux pathway and causing massive intracellular Cu accumulation. It is inferred that EC-6’s copper resistance primarily relies on Cu efflux rather than limiting copper uptake. Under non-stress conditions, the expression of copper homeostasis genes in the WT remained stable.

EC-6 displayed *ATP7* deficiency in its copper efflux system under non-copper stress, with RT-qPCR showing comp2884 and comp13468 downregulated by 11.57- and 10.66-fold, respectively (Table S5). Despite 2.48–3.88-fold upregulation of two *copA* transcripts, *ATP7* function was not compensated. The *PUT3* family exhibited dysregulated expression: comp5863 and comp839 were downregulated by 0.99- and 1.52-fold, while comp3495, comp6763, comp8290, comp5284, and comp5687 were upregulated by 1.23–3.74-fold (Table S5). This opposing expression pattern disrupted family-level coordination, resulting in a pre-stress phenotype of “*ATP7* downregulation-*PUT3* imbalance”. Under 0.5 mM copper stress, the WT copper efflux system was further activated: RT-qPCR revealed 4.29- and 6.74-fold increases in *copA* (comp478, comp1103) and a 6.26-fold increase in *ATP7* (comp3523) (Table S1). Most *PUT3* family members were upregulated, with only proline dehydrogenase showing mild (1.86–2.68-fold) downregulation (Table S1), indicating rapid restoration of copper homeostasis and proline cycling. In contrast, EC-6 showed delayed responses: *ATP7* (comp2884) remained downregulated by 2.65-fold (Table S3). While *copA* was upregulated by 2.22–3.67-fold, insufficient induction of comp2615 and comp478 (<0.3-fold) indicated efflux system impairment. The *PUT3* family exhibited further dysregulation: comp5863 was downregulated in both RNA-Seq and RT-qPCR, while comp839 and comp6763 were upregulated by 2–3-fold (Table S3), reducing coordination. Comparative analysis (EC-6 TY0.5 vs. WT TY0.5, Table S6) showed EC-6 *ATP7* (comp2884) and *copA* (comp2615) expression was 11.38- and 5.88-fold lower, respectively. This led to copper accumulation, elevated ROS levels, and suppressed spore production. Under 3 mM copper stress, EC-6 defects were exacerbated. The *PUT3* family shifted toward inhibition: RT-qPCR showed comp5284, comp3495, and comp5863 downregulated by 0.47-, 0.61-, and 2.14-fold, respectively (Table S4), contrasting with maintained or upregulated expression in the WT. Proline dehydrogenase exhibited conflicting changes: comp7977 was downregulated by 2.37-fold (RNA-Seq), while comp7926 was upregulated by 2.40-fold (Table S4), indicating metabolic pathway imbalance. Copper efflux system impairment worsened: despite 0.81–5.04-fold copA upregulation, *ATP7* components comp13468 and comp2884 were downregulated by 1.23- and 1.29-fold, respectively (Table S4). Comparative analysis (EC-6 TY3 vs. WT TY3, Table S2) showed EC-6 comp2884 expression was 10.55-fold lower. Collectively, high copper stress induced a triple defect in EC-6: “copper efflux impairment—*PUT3* inhibition—decreased proline degradation”, which correlated positively with reduced sporulation (Figure 3), suggesting these molecular events synergistically limit spore formation.

Partial inconsistencies exist between RT-qPCR and RNA-seq results, primarily due to technical platform differences (e.g., sensitivity, normalization methods), sample heterogeneity, primer specificity, and experimental errors [40,41,42]. Poor validation consistency is observed for low-expression genes [43]. In FEN1-knockdown cell models, some background genes showed no statistical difference in RT-qPCR, potentially related to cell cycle heterogeneity [44]. Additionally, RT-qPCR primers only recognize specific splice variants, whereas RNA-seq detects all transcripts, which may also cause discrepancies [45]. Multi-method cross-validation is required to ensure robust conclusions. Previous studies support the Cu-specific induction observed here: (i) In Bacillus subtilis, the *copA* operon promoter is up-regulated under high Cu but not by other metals, consistent with the 5.95- and 4.08-fold up-regulation detected in our RNA-seq [46]; transcriptomic data from GEORGE W. SUNDIN et al. [47] also show increased *copA* expression in the presence of Cu, in agreement with our results. (ii) Metagenomic and genomic characterization of the Avicennia marina rhizosphere microbiome indicate that Ni, Co or Cd marginally induce *ATP7*, whereas >0.2 mM Cu renders *ATP7* one of the most abundant heavy metal resistance genes [48], consistent with our observed *ATP7* up-regulation under 3 mM Cu. (iii) Conversely, Han et al. [49] reported that 0.2% calcium propionate (Ca^2+^ stress) significantly represses *MSB2* expression in Saccharomyces cerevisiae, presumably because Ca^2+^ does not trigger osmotic shock; the 2.05-fold up-regulation observed here under Cu stress may reflect the combined osmotic and oxidative insult imposed by Cu, which is known to activate the HOG-MAPK pathway. To our knowledge, no expression data for *PUT3*, *ALR*, *EIF6* or *VAPA* under heavy metal stress have been reported.

WGCNA can infer the function of unknown genes from known ones and identify additional important genes that are similar based on their correlation with other genes [50,51,52]. In this study, WGCNA was applied to analyze transcriptome data from six samples (WT and EC-6) of *P. janthinellum* under copper stress, combined with physiological and biochemical experimental results. All genes were grouped into 12 modules based on their expression patterns. Among these, two modules showed the highest positive correlation with copper stress and also exhibited strong positive correlations with ROS and CAT traits. One module was associated with copper-manganese interaction traits, while most other modules showed negative correlations with the measured traits. A total of 116 hub genes were identified; these genes occupy central positions in module network regulation and exhibit high connectivity with other genes. Database comparisons revealed that most of these hub genes are associated with energy metabolism, cellular components, and transporters, which is consistent with the expected results. Jikai Zong et al. used transcriptome-based WGCNA to uncover the mechanisms underlying drought-tolerance differences in sweet potato, providing important insights into the molecular basis of sweet potato drought resistance [53]. Jingwen Yang et al. employed transcriptome-based WGCNA to reveal the regulated metabolic flux between flower color and floral scent in Narcissus, indicating a potential trade-off between pigments and scent during single-flower development and evolutionary processes [54]. Researchers worldwide have combined WGCNA with machine learning algorithms to identify and validate shared genes and key pathways in endometriosis and endometriosis-associated ovarian cancer [55], explore hub genes in aging brains [56], predict diagnostic gene biomarkers for patients with diabetic nephropathy [57], and search for diagnostic and prognostic gene expression patterns [58]. Subsequent studies could integrate the WGCNA of *Penicillium janthinellum* with machine learning algorithms and bioinformatics techniques to identify key genes governing copper resistance in this genus.

## 4. Materials and Methods

### 4.1. Fungal Strains, Media, and Growth Conditions

The WT strain used in this study was *P. janthinellum* GXCR [17]. Mutant EC-6 [17] was generated by subjecting the WT strain to combined ultraviolet (UV) irradiation and ethyl methanesulfonate (EMS) mutagenesis, while mutant UC-8 [17] was obtained by subjecting the wild-type strain to combined UV irradiation and ^60^Co γ-ray radiation mutagenesis. Both mutants (EC-6 and UC-8) exhibited lower copper resistance compared to the WT strain, and no strains with higher copper resistance than the WT strain were identified. The following media were used for fungal cultivation: Conventional potato dextrose agar (PDA): 40 g of PDA powder (HuanKai Microbial Technology Co., Ltd., Guangzhou, China) was added to a beaker containing deionized water. After slow stirring until complete dissolution, the volume of the solution was adjusted to 1 L, followed by aliquoting. The medium was autoclaved at 121 °C for 20 min. Agar-free liquid potato dextrose broth (PDB): 24 g of PDB powder (HuanKai Microbial Technology Co., Ltd., Guangzhou, China) was weighed into a beaker, and deionized water was added. After slow stirring until complete dissolution, the volume was adjusted to 1 L, followed by aliquoting and autoclaving at 121 °C for 20 min. TYA and TYB media (1 L preparation): 2 g of sodium gluconate and 1 g of yeast extract were weighed, and deionized water was added to reach a final volume of 1 L. The pH of the solution was adjusted to 7.0. For TYA medium, agar powder was added at a concentration of 2% (*w*/*v*) prior to aliquoting and autoclaving at 121 °C for 20 min. Fungal cultures were maintained at 32 °C.

### 4.2. Morphological Observation of Wild-Type and Mutant Strains

The spore suspension was removed from the −80 °C freezer. In a laminar flow hood, 1 μL of the spore suspension was transferred using a pipette and inoculated onto potato dextrose agar (PDA) plates. The plates were incubated upside down at 32 °C for one week. Subsequently, in the laminar flow hood, a sterile toothpick was used to inoculate the center of fresh PDA plates, which were then incubated at 32 °C for another week. After one week of growth on PDA plates, the *P. janthinellum* colonies were transferred to a laminar flow hood. A 10 mL spore wash solution was added to collect the spores, and the concentration of the spore suspension was adjusted to 1 × 10^6^ spores/mL using an enzyme-linked immunosorbent assay (ELISA) reader (Agilent BioTek Epoch, Santa Clara, CA, USA). The adjusted spore suspension was aliquoted into sterile Eppendorf tubes and stored at 4 °C for subsequent use. A small volume of the spore suspension (1 × 10^6^ spores/mL) was inoculated onto PDA and TYA plates [59] supplemented with different concentrations of CuSO_4_, MnCl_2_, or proline. The inoculated plates were incubated at 32 °C for 7–10 days, and the morphology of the strains under different culture conditions was observed. The copper tolerance of the wild-type and mutant strains were also determined.

### 4.3. Determination of Spore Germination Rate

Spore suspensions of the wild-type and mutant strains (1 × 10^6^ spores/mL) were separately inoculated into 100 mL volumes of potato dextrose broth (PDB) and TYB medium supplemented with 0, 0.5, 1, 2, 3, 4, or 5 mM Cu^2+^ or Cr^6+^. Cultivation was carried out at 32 °C with a shaking speed of 200 rpm. At 8 h, 12 h, 16 h, and 24 h post-inoculation, aliquots of the spore suspensions were collected. Spores were counted under a microscope, and the germination rate was calculated using the following formula: Spore germination rate (per field of view) = (Number of germinated spores/Total number of spores) × 100%.

### 4.4. Determination of Reactive Oxygen Species (ROS) and Antioxidant Enzyme Activities of Strains Under Copper Stress

Reactive oxygen species (ROS) within bacterial cells were detected using the Solarbio Reactive Oxygen Species Assay Kit (Beijing Solarbio Technology Co., Ltd., Beijing, China), while superoxide dismutase (SOD) within bacterial cells was detected using the Superoxide Dismutase Assay Kit (Beijing Solarbio Technology Co., Ltd., Beijing, China). Reference [60] was used to determine catalase (CAT) activity within bacterial cells, while the guaiacol method was referenced for peroxidase (POD) activity measurement [60].

### 4.5. The Accumulation of Different Heavy Metals in the Cell Was Determined

Spore suspensions of the wild-type and mutant strains (concentration: 1 × 10^6^ spores/mL) were inoculated into conical flasks containing TYB medium at a 1% (*v*/*v*) inoculum volume, and cultivated in a shaking incubator (32 °C, 200 rpm) for 3 days. To prepare the heavy metal ion elution solution: 70 mL of 1 M Tris buffer (pH = 7.5; Solarbio Science & Technology Co., Ltd., Beijing, China) and 30 mL of 0.5 M EDTA (pH = 8.0; Solarbio Science &Technology Co., Ltd., Beijing, China) were measured. Deionized water was added to adjust the final volume to 1000 mL, and the solution was autoclaved at 121 °C for 20 min. Within a laminar flow hood (Suzhou Purification Equipment Co., Ltd., Suzhou, China), mycelial pellets were filtered using sterile gauze. The mycelial pellets were washed multiple times with the heavy metal ion elution solution, followed by three rinses with deionized water. The washed mycelia were collected into a 50 mL centrifuge tube and dried in an electric heating oven at 50 °C; the final dry weight was recorded. Subsequently, the dried mycelial samples were subjected to microwave digestion, and the contents of copper (Cu), manganese (Mn), chromium (Cr), cadmium (Cd), and lead (Pb) in the fungal biomass were determined using flame atomic absorption spectroscopy.

### 4.6. Transcriptome Detection

WT and mutant spore suspensions (concentration: 1 × 10^6^ spores/mL) were separately inoculated into conical flasks containing TYB medium at a 1% (*v*/*v*) inoculum volume. After incubation in a shaking incubator (32 °C, 200 rpm) for 3 days, the fungal cells were washed several times with heavy metal ion eluate, followed by rinsing with deionized water. The washed cells were then treated with 5–10 volumes of RNAlater Solution. Samples were incubated overnight at 4 °C and subsequently stored at −80 °C. Total RNA was extracted from six samples (wild-type strain WT and mutant strain EC-6 cultured in TYB medium with copper concentrations of 0, 0.5, and 3 mM) using a plant RNA extraction kit (Jiangsu Kangwei Century Biotechnology Co., Ltd., Nanjing, China). Reverse transcription was performed using the HiScript II Reverse Transcriptase Kit (Novogene Bio-Technology Co., Ltd., Nanjing, China). The resulting reverse transcription products were subjected to library preparation, and sequencing was conducted on an Illumina HiSeq 2000 sequencer (Illumina, San Diego, CA, USA). RT-qPCR was performed using the AceQ qPCR SYBR Green Master Mix Kit (Novogene Bio-Technology Co., Ltd., Nanjing, China). The transcriptomic data used in this study were derived from the work of Jian Xu et al. (2015) [17]. Sequencing was performed with Solexa RNA paired-end technology to generate a large set of transcript reads, which were filtered through quality preprocessing to yield valid reads. All effective transcript sequences have been deposited in the GenBank nucleotide database (http://www.ncbi.nlm.nih.gov/nuccore/ (accessed on 15 July 2014)) under accession numbers GBSP01000001 to GBSQ01013184. The valid reads from the six fungal samples were pooled and de novo assembled with Trinity (version 2.6.0) using a paired-end strategy, followed by redundancy removal to produce the final Unigene set.

### 4.7. RT-qPCR Validation

To validate transcriptome sequencing data, a set of copper-resistance-related differentially expressed genes was selected. Total RNA was reverse-transcribed into cDNA using the HiScript II Reverse Transcriptase kit (Vazyme Biotech Co., Ltd., Nanjing, China). Gene-specific primers were designed with Primer Premier 5.0 (Table S14), and α-tubulin served as the internal reference. RT-qPCR was performed on an ABI StepOnePlus (Thermo Fisher Scientific, Waltham, MA, USA) instrument using the SYBR Green method with AceQ qPCR SYBR Green Master Mix (Vazyme Biotech Co., Ltd., Nanjing, China). PCR products were examined on 2.0% TAE-agarose gels. RT-qPCR validation was performed using the same *P. janthinellum* WT strain and EC-6 mutant as the RNA-seq analysis, the same TYB medium (mineral salt liquid medium), and copper concentrations of 0 mM, 0.5 mM, and 3 mM, completely matching the RNA-seq experimental design. Relative expression levels under the same treatments used for RNA-seq were calculated by the 2^^ΔΔCt^ method [61].

### 4.8. WGCNA

WGCNA was performed using the WGCNA package (version 1.63) in R software (Windows version R-3.4.0), with reference to the official WGCNA network resource (https://cran.r-project.org/src/contrib/Archive/WGCNA/ (accessed on 24 July 2025)). Co-expression modules were obtained using the automatic network construction function, and the correlation between each module and the treatment was calculated to derive the module eigengene values. The analysis results were visualized using Cytoscape software (version 3.6.1 for Windows 64-bit) for network construction. The specific analysis workflow was as follows: (1) Scale-free distribution of gene relationships: The correlation coefficients between gene expression levels were raised to the nth power, gradually aligning the distribution of correlation values with a scale-free distribution. (2) Gene grouping into co-expression modules: Genes were classified based on their expression patterns, with genes exhibiting similar expression patterns grouped into a single module. (3) Assignment of genes to modules: Genes with similar expression patterns were assigned to the same co-expression module. (4) Identification of target modules: Functional enrichment analysis was conducted for each module; correlations between modules and specific sample traits were examined; composite expression values for gene groups were calculated using mathematical methods; and module correlations with selected samples were evaluated. (5) Identification of hub genes within modules: Hub genes are typically key regulatory factors, such as transcription factors.

### 4.9. Statistical Analysis

Data were processed with Microsoft Excel 2021 to calculate means and standard errors of biological replicates. ANOVA and correlation analyses were performed with SPSS, version 26.0. Heatmaps were generated with TBtools, version 2.096 [62]. Significance was assessed by *t*-tests at *p* ≤ 0.05. Values are presented as means ± SE of three independent biological samples.

## 5. Conclusions

Exogenous proline alleviates copper stress by reducing cellular osmotic pressure, promoting strain growth and sporulation. Manganese ions enhance copper resistance by competing for transport channels to decrease intracellular copper content, while manganese co-stress further lowers intracellular copper content by increasing the activity of antioxidant enzymes (POD, SOD, CAT) and altering membrane permeability. Copper stress initially increases antioxidant enzyme activity, but high-concentration stress leads to a rapid decline in their activity, triggering ROS accumulation and cell damage; intracellular copper content shows a trend of first increasing then decreasing, which is presumably associated with active copper efflux and ROS accumulation. WT efficiently activates copper efflux/homeostasis pathways (*copA*, *ATP7*) and proline metabolism pathways (*PUT3*, proline dehydrogenase) under copper stress. In contrast, EC-6 exhibits *ATP7* expression deficiency, resulting in failed copper efflux, ROS accumulation, and sporulation inhibition; its *PUT3* family genes display disordered expression, disrupting proline metabolism and spore formation processes. This study has certain limitations: all screening of copper resistance-related genes and construction of regulatory networks were based on transcriptome sequencing and bioinformatics analysis, and the functions of key candidate genes have not been validated through molecular genetic experiments, such as gene knockout. This study provides novel insights into the copper resistance mechanisms of *P. janthinellum*, offers potential targets to explore the cross-species conservation and application potential of key genes, and contributes to the bioremediation of heavy metal pollution. This research holds important theoretical and practical value in the field of microbial heavy metal resistance. For example, after expressing the key differentially regulated genes (including *PSTPIP1*, *MSB2*, etc.) identified in this study in wild-type industrial brewing yeast, the yeast exhibited enhanced tolerance.

## Figures and Tables

**Table 1:** The copper tolerance of strains under copper stress.

Medium	WT (mM)	UC-8 (mM)	EC-6 (mM)
PDA	1000	60	40
PDB	120	8	7
TYA	800	25	20
TYB	100	5	4

## Data Availability

The original contributions presented in this study are included in the article/Supplementary Material. Further inquiries can be directed to the corresponding authors.

## Supplementary Materials

The following supporting information can be downloaded at: https://www.mdpi.com/article/10.3390/ijms27073290/s1.

## References

[1] Yu Q., Li W., Zhang N. (2024). Present pollution status and safe utilization technologies of heavy metal-polluted farmland soil: A review. Soils.

[2] Zaynab M., Al-Yahyai R., Ameen A., Sharif Y., Ali L., Fatima M., Khan K.A., Li S. (2022). Health and environmental effects of heavy metals. J. King Saud Univ. Sci..

[3] Li M., Rao J., Liu X., Huang S., Xiao R., Yu K., Wang D., Wang Y., Yu Q., Zhang Z. (2025). Current status and prospects of comprehensive utilization of copper smelting slag. China Min. Mag..

[4] Nie G., He R., Tang Y. (2015). The biological toxicity of copper oxide nanoparticles and its toxicology mechanisms. Chin. Bull. Life Sci..

[5] Sun F., Xu Z., Fan L. (2021). Response of heavy metal and antibiotic resistance genes and related microorganisms to different heavy metals in activated sludge. J. Environ. Manag..

[6] Yang P., Condrich A., Lu L., Scranton S., Hebner C., Sheykhhasan M., Ali M.A. (2024). Genetic Engineering in Bacteria, Fungi, and Oomycetes, Taking Advantage of CRISPR. DNA.

[7] Wei M.K. (2003). Study on the Resistance of *Penicillium* sp. GXCR1 to Toxic Heavy Metal Salts and Its Genetic Transformation System. Master’s Thesis.

[8] Zhou Y., Huang X., Huang G., Bai X., Li Y. (2008). Cu and Fe bioleaching in low-grade chalcopyrite and bioleaching mechanisms using Penicillium janthinellum strain GXCR. Chin. J. Biotechnol..

[9] Su H., Ying T.H., Ling L., Ken W.M., Na W., Zhi L.Y. (2006). Characteristics of resistance of Penicillium janthinellum strain GXCR to Cu, Al and Zn, and its Cu biomineralization. J. Guangxi Agric. Biol. Sci..

[10] Raffa N., Osherov N., Keller N.P. (2019). Copper utilization, regulation, and acquisition by Aspergillus fumigatus. Int. J. Mol. Sci..

[11] Ben Ghorbal S.K., Maalej L., Ouzari I.-H., Chatti A. (2023). Implication of Mn-cofactored superoxide dismutase in the tolerance of swarmer Pseudomonas aeruginosa to polymixin, ciprofloxacin and meropenem antibiotics. World J. Microbiol. Biotechnol..

[12] Koç E., Karayiğit B. (2024). The protective role of exogenous proline in pepper callus exposed to long-term cold stress. Bot. Serbica.

[13] Kavi Kishor P.B., Suravajhala P., Rathnagiri P., Sreenivasulu N. (2022). Intriguing role of proline in redox potential conferring high temperature stress tolerance. Front. Plant Sci..

[14] Zhao M., Wang C.J., Zhou E.X., Shu C.W. (2019). The Latest Research Progress on Genetic Transformation System of Filamentous Fungi.

[15] Sassa T., Nukina M., Sugiyama T., Yamshita K. (1983). Monilidiols, characteristic and bioactive metabolites of benomyl-resistant strains of Monilinia fructicola. Agric. Biol. Chem..

[16] Balabanova L.A., Shkryl Y.N., Slepchenko L.V., Yugay Y.A., Gorpenchenko T.Y., Kirichuk N.N., Khudyakova Y.V., Bakunina I.Y., Podvolotskaya A.B., Bulgakov V.P. (2019). Development of host strains and vector system for an efficient genetic transformation of filamentous fungi. Plasmid.

[17] Xu J., Chen G.-L., Sun X.-Z., Fan X.-W., You-Zhi L. (2015). Paths and determinants for Penicillium janthinellum to resist low and high copper. Sci. Rep..

[18] Shapiro R.S., Robbins N., Cowen L.E. (2011). Regulatory circuitry governing fungal development, drug resistance, and disease. Microbiol. Mol. Biol. Rev..

[19] Zhao J., Csetenyi L., Gadd G.M. (2020). Biocorrosion of copper metal by Aspergillus niger. Int. Biodeterior. Biodegrad..

[20] Yao Z., Zou C., Zhou H., Wang J., Lu L., Li Y., Chen B. (2013). Δ1-pyrroline-5-carboxylate/glutamate biogenesis is required for fungal virulence and sporulation. PLoS ONE.

[21] Evan D., Bottrell A.D., Kari N. (2021). Identifying the Effects of Reactive Oxygen Species on Mitochondrial Dynamics and Cytoskeleton Stability in *Dictyostelium discoideum*. Cells.

[22] Shi H.T. (2016). Laboratory Manual of Plant Stress Physiology.

[23] Chen X., Lu Z., Chen Y., Wu R., Luo Z., Lu Q., Guan N., Chen D. (2021). Deletion of the *MBP1* Gene, Involved in the Cell Cycle, Affects Respiration and Pseudohyphal Differentiation in *Saccharomyces cerevisiae*. Microbiol. Spectr..

[24] Liu F., Zhao Y., Wang X., Wang B., Xiao F., He K. (2023). Physiological response and drought resistance evaluation of Gleditsia sinensis seedlings under drought-rehydration state. Sci. Rep..

[25] Fu X., Zhang Y., Jiang W., Monnot A.D., Bates C.A., Zheng W. (2014). Regulation of copper transport crossing brain barrier systems by Cu-ATPases: Effect of manganese exposure. Toxicol. Sci..

[26] Chen X., Yang X., Xie J., Ding W., Li Y., Yue Y., Wang L. (2020). Biochemical and Comparative Transcriptome Analyses Reveal Key Genes Involved in Major Metabolic Regulation Related to Colored Leaf Formation in Osmanthus fragrans ‘Yinbi Shuanghui’ during Development. Biomolecules.

[27] Huang J., Zhou Q. (2022). Gene Biomarkers Related to Th17 Cells in Macular Edema of Diabetic Retinopathy: Cutting-Edge Comprehensive Bioinformatics Analysis and In Vivo Validation. Front. Immunol..

[28] Hollender C.A., Kang C., Omar D., Aviva G., Matthews B.F., Janet S., Nadim A., Liu Z. (2014). Floral Transcriptomes in Woodland Strawberry Uncover Developing Receptacle and Anther Gene Networks. Plant Physiol..

[29] Marcussen T., Sandve S.R., Heier L., Spannagl M., Pfeifer M., Jakobsen K.S., Wulff B.B.H., Steuernagel B., Mayer K.F.X., The International Wheat Genome Sequencing Consortium (2014). Ancient hybridizations among the ancestral genomes of bread wheat. Science.

[30] Vasantha N., Freese E. (1979). The role of manganese in growth and sporulation of Bacillus subtilis. Microbiology.

[31] Erzsébet S., Kolláth I.S., Erzsébet F., Vivien B., Michel F., Béla K., Kubicek C.P., Levente K. (2021). Carbon-Source Dependent Interplay of Copper and Manganese Ions Modulates the Morphology and Itaconic Acid Production in *Aspergillus terreus*. Front. Microbiol..

[32] Cizewski C.V., Mei Y., Hall M.D. (2005). Manganese transport and trafficking: Lessons learned from Saccharomyces cerevisiae. Eukaryot. Cell.

[33] Fan J.J., Yuan Y.H. (2015). Laboratory Manual of Plant Physiology.

[34] Li F., Chen S., Liu M., Chen M., Li X., Liu B. (2022). Transcriptome analysis of copper resistance in Lysobacter soli strian RCu6. Chin. J. Eco-Agric..

[35] Okolie C.U., Mbaukwu A.O., Anukwuorji C.A., Okereke C.N. (2024). Immobilization of Heavy Metals in Contaminated Soils Using Phosphate-Solubilizing Fungi: Mechanisms and Advantages. Nat. Resour. Conserv..

[36] Sun W., Zhao L., Zhou J., Feng H., Zhang Y., Feng Z., Zhu H., Wei F. (2024). VdP5CDH is involved in melanin formation, stress resistance and play a regulatory role in virulence of Verticillium dahliae. Front. Microbiol..

[37] Idol R.A., Dinauer M.C. (2017). Aspergillus fumigatus copper export machinery and reactive oxygen intermediate defense counter host copper-mediated oxidative antimicrobial offense. Cell Rep..

[38] Fan C., Zhang L., Fu H., Liu C., Li W., Cheng Q., Zhang H., Jia S., Zhang Y. (2020). Enterotypes of the gut microbial community and their response to plant secondary compounds in plateau pikas. Microorganisms.

[39] Valverde R.H.F., Lowe J. (2024). Protein Kinases in Copper Homeostasis: A Review on Cu^+^-ATPase Modulation. Kinases Phosphatases.

[40] Everaert C., Luypaert M., Maag J.L., Cheng Q.X., Dinger M.E., Hellemans J., Mestdagh P. (2017). Benchmarking of RNA-sequencing analysis workflows using whole-transcriptome RT-qPCR expression data. Sci. Rep..

[41] Coenye T. (2021). Do results obtained with RNA-sequencing require independent verification?. Biofilm.

[42] Luo H., Zhu Y., Wang J., Wang Y., Wei L. (2023). Comprehensive profile and contrastive analysis of circular RNA expression in cervical squamous carcinoma and adenocarcinoma. PeerJ.

[43] Sha Y., Phan J.H., Wang M.D. (2015). Effect of low-expression gene filtering on detection of differentially expressed genes in RNA-seq data. Proceedings of the 2015 37th Annual International Conference of the IEEE Engineering in Medicine and Biology Society.

[44] Su Q., Wu J., Du J.H., Yang F., Yuan P.Y., Liu S.B., Qiu X.Q. (2019). Validation of RNA-seq Results in FEN1-Knockdown Cells by qRT-PCR. Chongqing Med. J..

[45] Zhu B., Yan Y. (2021). Application of single-cell RNA sequencing technology in the study of skeletal system biology. Chin. J. Tissue Eng. Res..

[46] Radford D.S., Kihlken M.A., Borrelly G.P., Harwood C.R., Le Brun N.E., Cavet J.S. (2003). CopZ from Bacillus subtilis interacts in vivo with a copper exporting CPx-type ATPase CopA. FEMS Microbiol. Lett..

[47] Águila-Clares B., Castiblanco L.F., Quesada J.M., Penyalver R., Carbonell J., López M.M., Marco-Noales E., Sundin G.W. (2018). Transcriptional response of Erwinia amylovora to copper shock: In vivo role of the copA gene. Mol. Plant Pathol..

[48] Andrea M.-G., Ziv A., Vivian B.-V., Javier V. (2022). Metagenomic and genomic characterization of heavy metal tolerance and resistance genes in the rhizosphere microbiome of Avicennia germinans in a semi-arid mangrove forest in the tropics. Mar. Pollut. Bull..

[49] Ye H., Li X., Zhang X., Xiao Z., Xu C., Huang C. (2021). Antimicrobial mechanism of calcium propionate on Saccharomyces cerevisiae based on transcriptomics analysis. Microbiol. China.

[50] Langfelder P., Zhang B., Horvath S. (2008). Defining clusters from a hierarchical cluster tree: The Dynamic Tree Cut package for R. Bioinformatics.

[51] Barabasi A.L., Oltvai Z.N. (2004). Network biology: Understanding the cell’s functional organization. Nat. Rev. Genet..

[52] Langfelder P., Horvath S. (2008). WGCNA: An R package for weighted correlation network analysis. BMC Bioinform..

[53] Zong J., Chen P., Luo Q., Gao J., Qin R., Wu C., Lv Q., Zhao T., Fu Y. (2023). Transcriptome-based WGCNA analysis reveals the mechanism of drought resistance differences in sweetpotato (*Ipomoea batatas* (L.) Lam.). Int. J. Mol. Sci..

[54] Yang J., Ren Y., Zhang D., Chen X., Huang J., Xu Y., Aucapiña C.B., Zhang Y., Miao Y. (2021). Transcriptome-based WGCNA analysis reveals regulated metabolite fluxes between floral color and scent in *Narcissus tazetta* flower. Int. J. Mol. Sci..

[55] Du Y., Hu Y., Zhang F., Guan Y., Sheng Y. (2023). Identification and validation of shared genes and key pathways in endometriosis and endometriosis-associated ovarian cancer by weighted gene co-expression network analysis and machine learning algorithms. J. Obstet. Gynaecol. Res..

[56] Chai K., Liang J., Zhang X., Cao P., Chen S., Gu H., Ye W., Liu R., Hu W., Peng C. (2021). Application of Machine Learning and Weighted Gene Co-expression Network Algorithm to Explore the Hub Genes in the Aging Brain. Front. Aging Neurosci..

[57] Gao Q., Jin H., Xu W., Wang Y. (2023). Predicting diagnostic gene biomarkers in patients with diabetic kidney disease based on weighted gene co expression network analysis and machine learning algorithms. Medicine.

[58] Grima N., Liu S., Southwood D., Henden L., Smith A., Lee A., Rowe D.B., D’Silva S., Blair L.P., Williams K.L. (2023). RNA sequencing of peripheral blood in amyotrophic lateral sclerosis reveals distinct molecular subtypes: Considerations for biomarker discovery. Neuropathol. Appl. Neurobiol..

[59] Mergeay M., Nies D., Schlegel H., Gerits J., Charles P., Van Gijsegem F. (1985). Alcaligenes eutrophus CH34 is a facultative chemolithotroph with plasmid-bound resistance to heavy metals. J. Bacteriol..

[60] Wang X.K. (2012). Principles and Techniques of Plant Physiological and Biochemical Experiments.

[61] Schmittgen T.D., Livak K.J. (2008). Analyzing real-time PCR data by the comparative CT method. Nat. Protoc..

[62] Chen C., Chen H., Zhang Y., Thomas H.R., Frank M.H., He Y., Xia R. (2020). TBtools: An integrative toolkit developed for interactive analyses of big biological data. Mol. Plant.

