# Supplementary material for: Transcriptome-Based WGCNA Reveals Hub Genes Involved in Copper Resistance of Penicillium janthinellum GXCR
Source: Int J Mol Sci. 2026 Apr 4;27(7):3290. doi: 10.3390/ijms27073290 (PMC13073840; doi:10.3390/ijms27073290)
Supplement: Supplementary file 1 [file ijms-27-03290-s001.zip › ijms-4172635-supplementary.pdf]

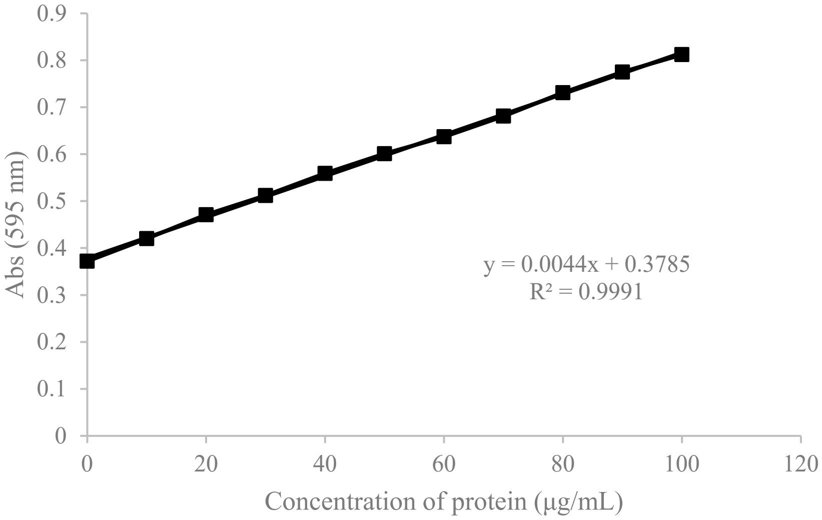

Figure S1: The standard curve of protein BSA

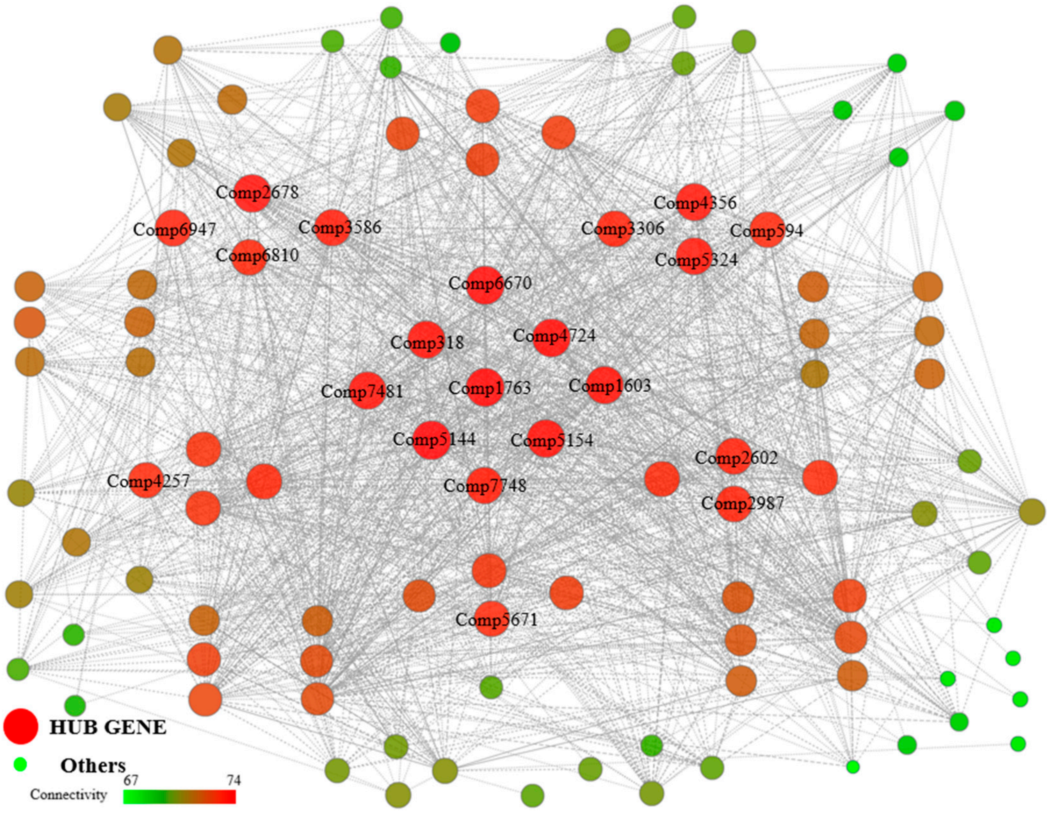

Figure S2: The correlation network of MEgreen module.

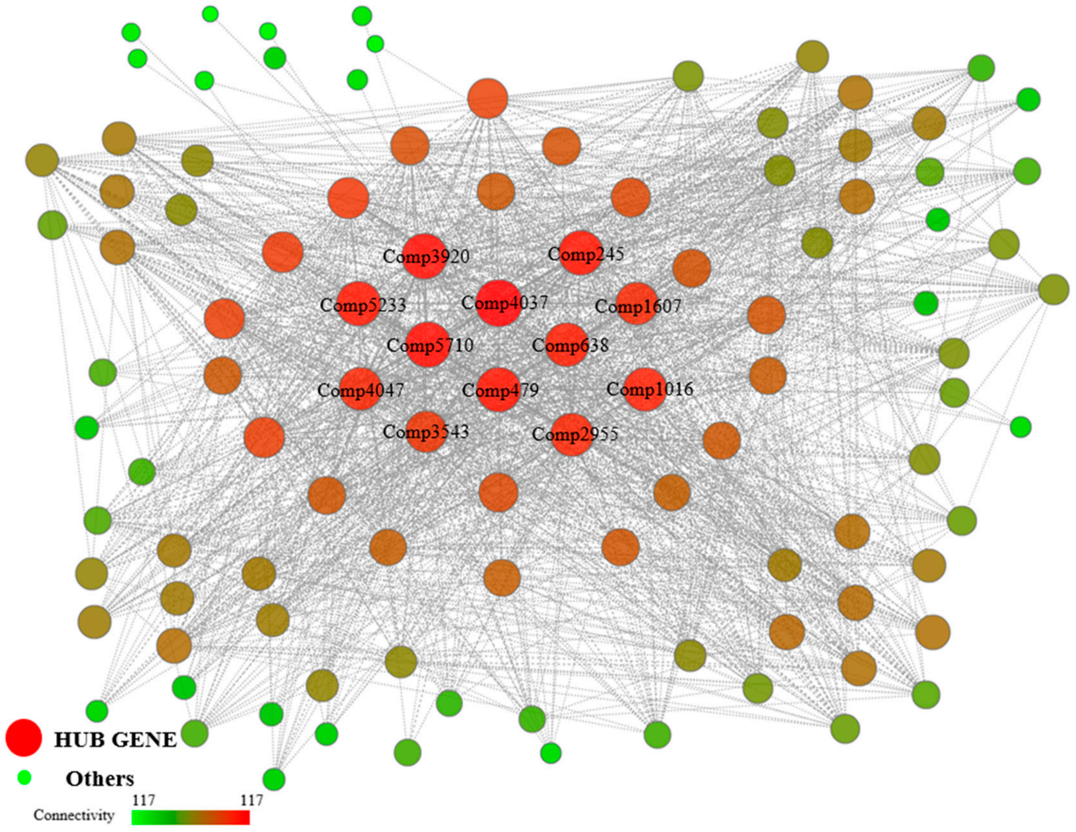

Figure S3: The correlation network of MEturquoise module.

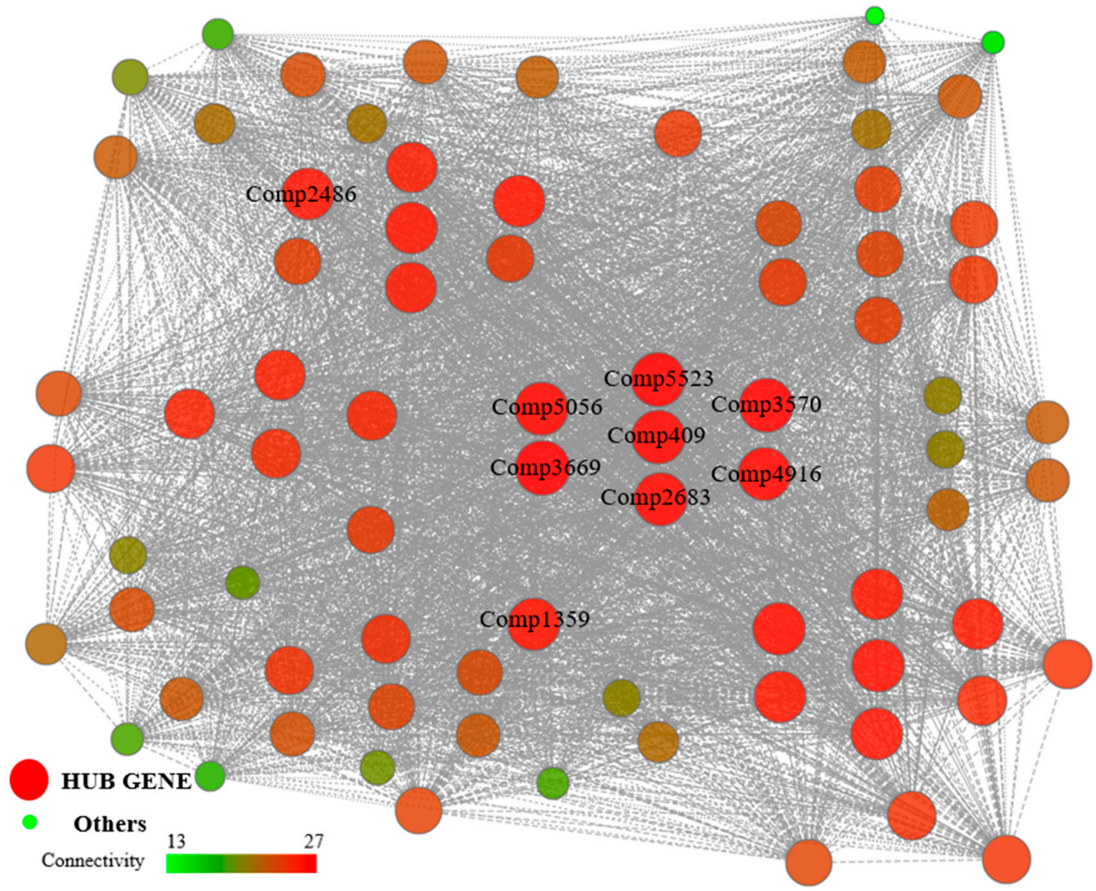

Figure S4: The correlation network of MEgreenyellow module.

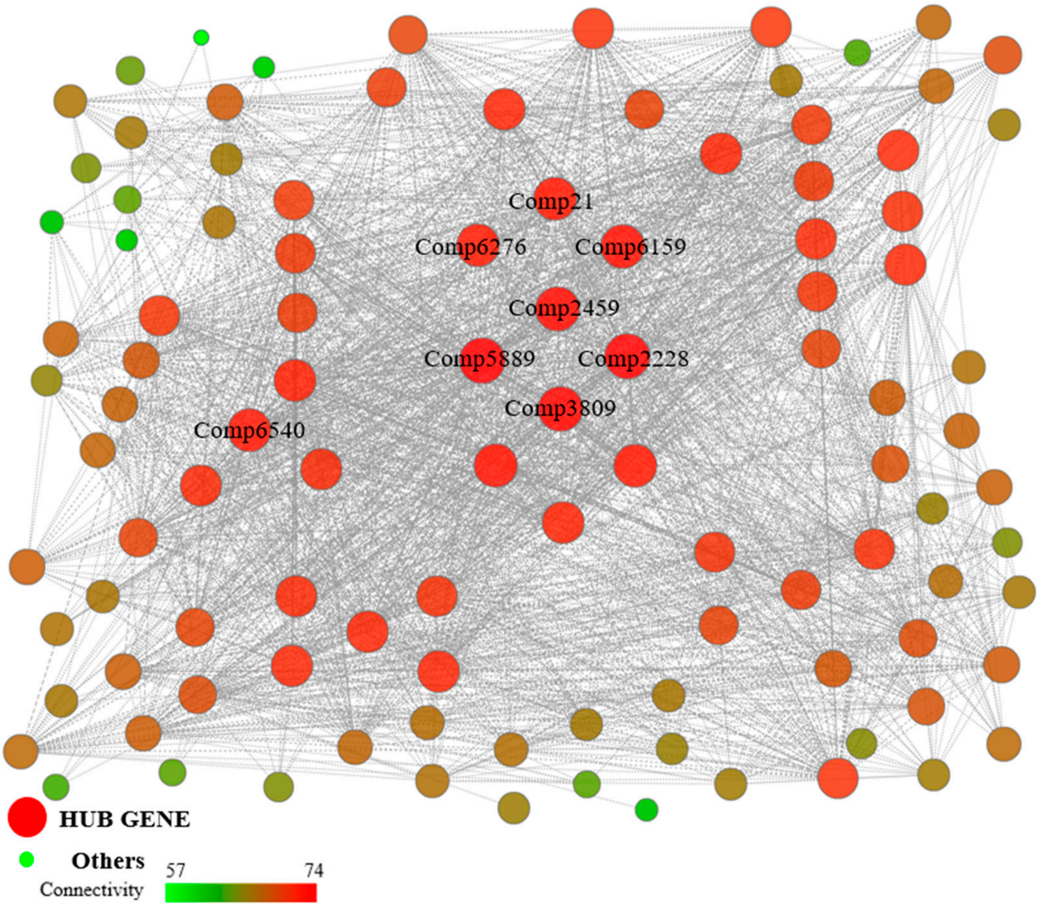

Figure S5: The correlation network of MEmidnightblue module.

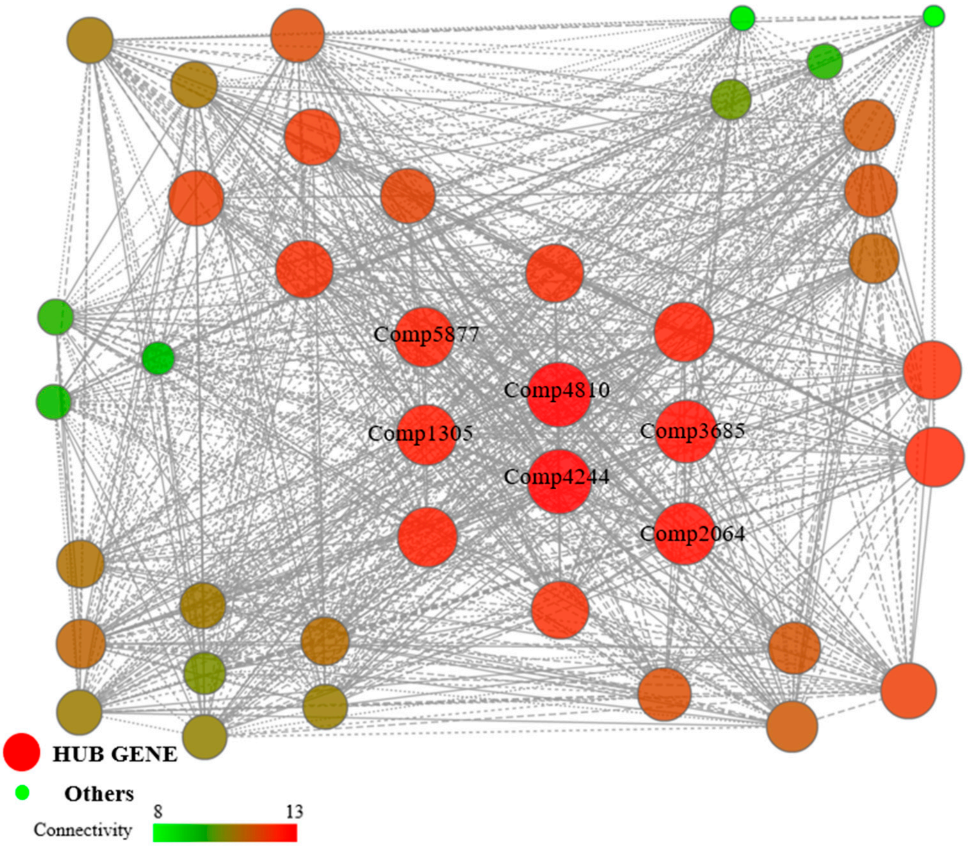

Figure S6: The correlation network of MEgrey module.

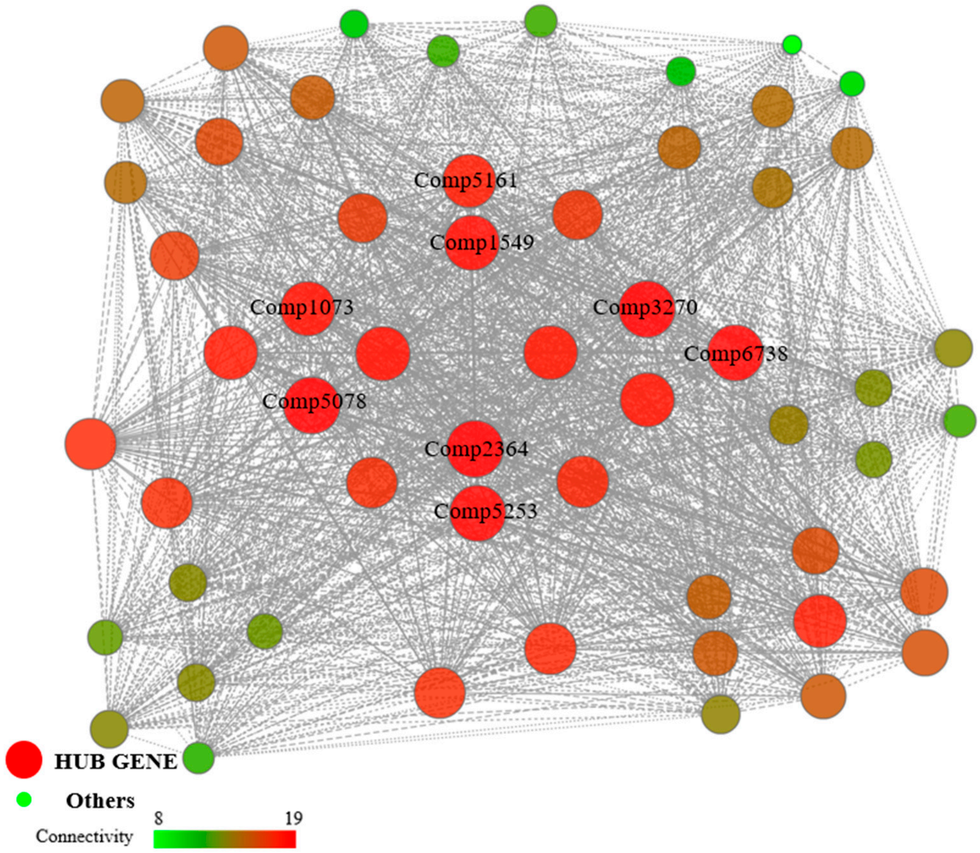

Figure S7: The correlation network of MEcyan module.

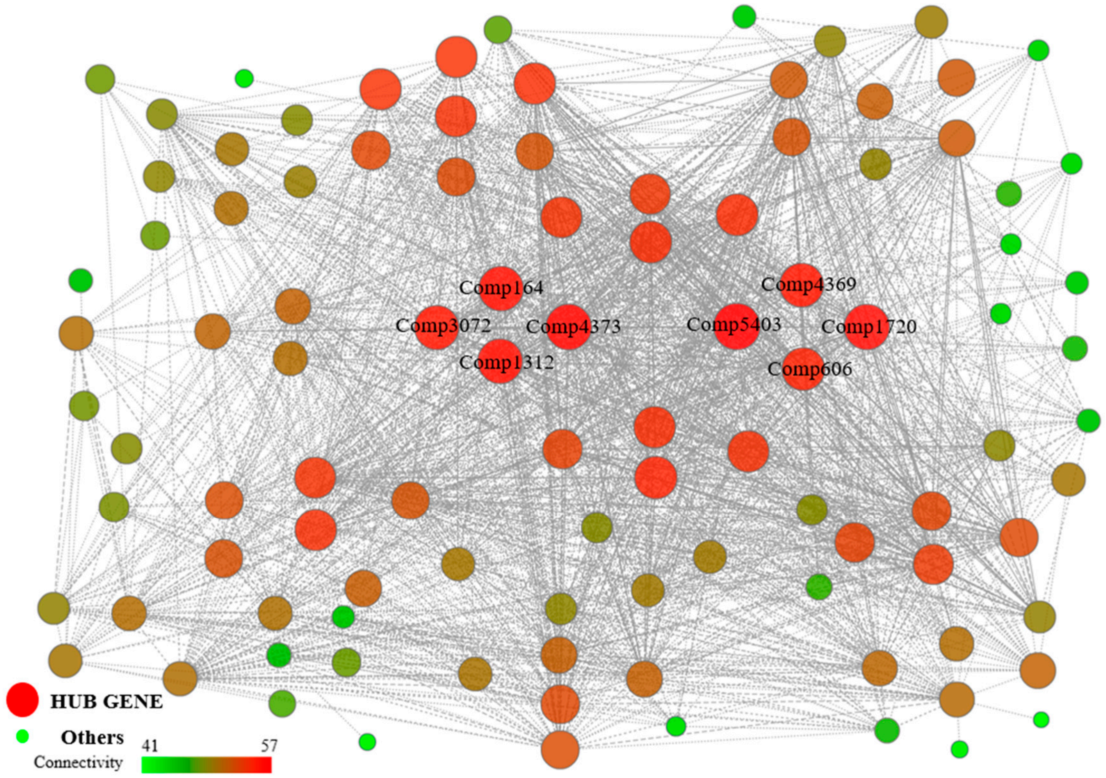

Figure S8: The correlation network of MEsalmon module.

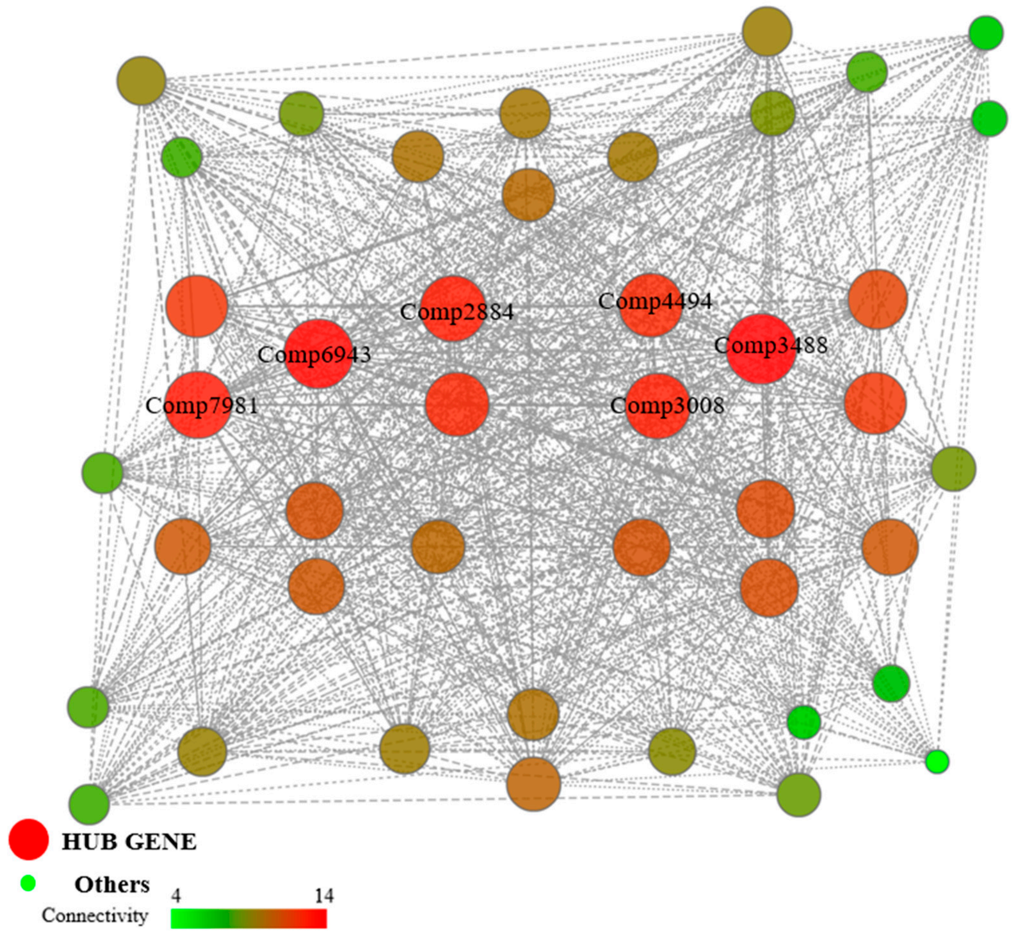

Figure S9: The correlation network of MElightcyan module.

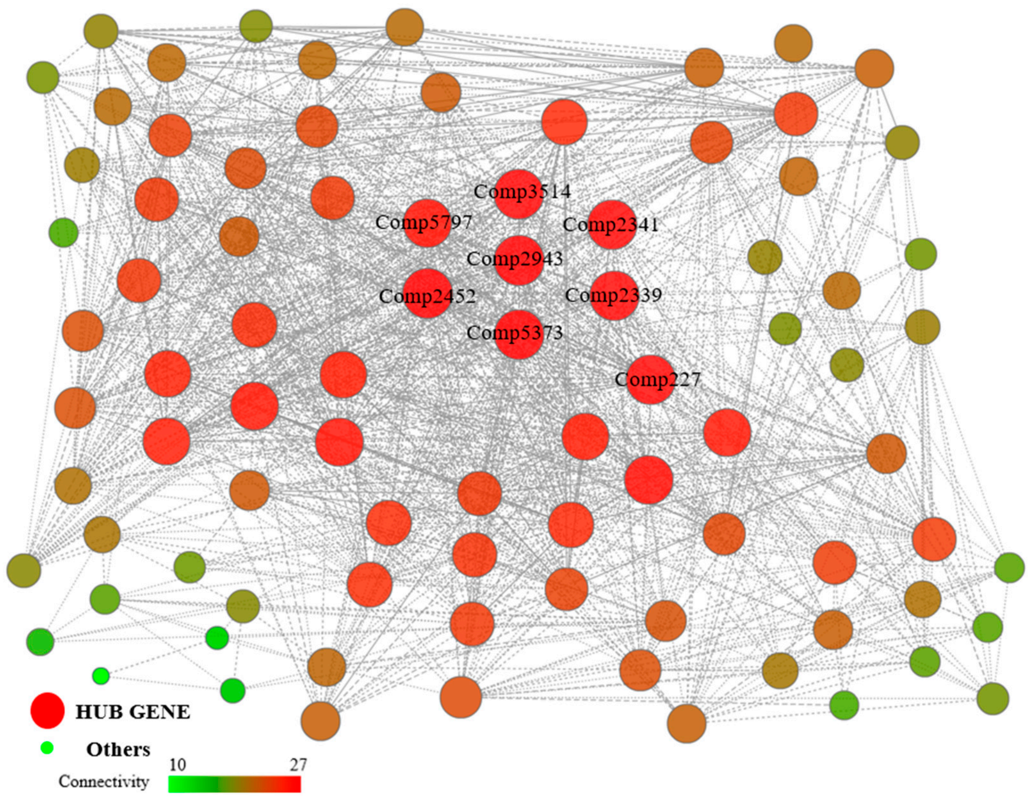

Figure S10: The correlation network of MEpink module.

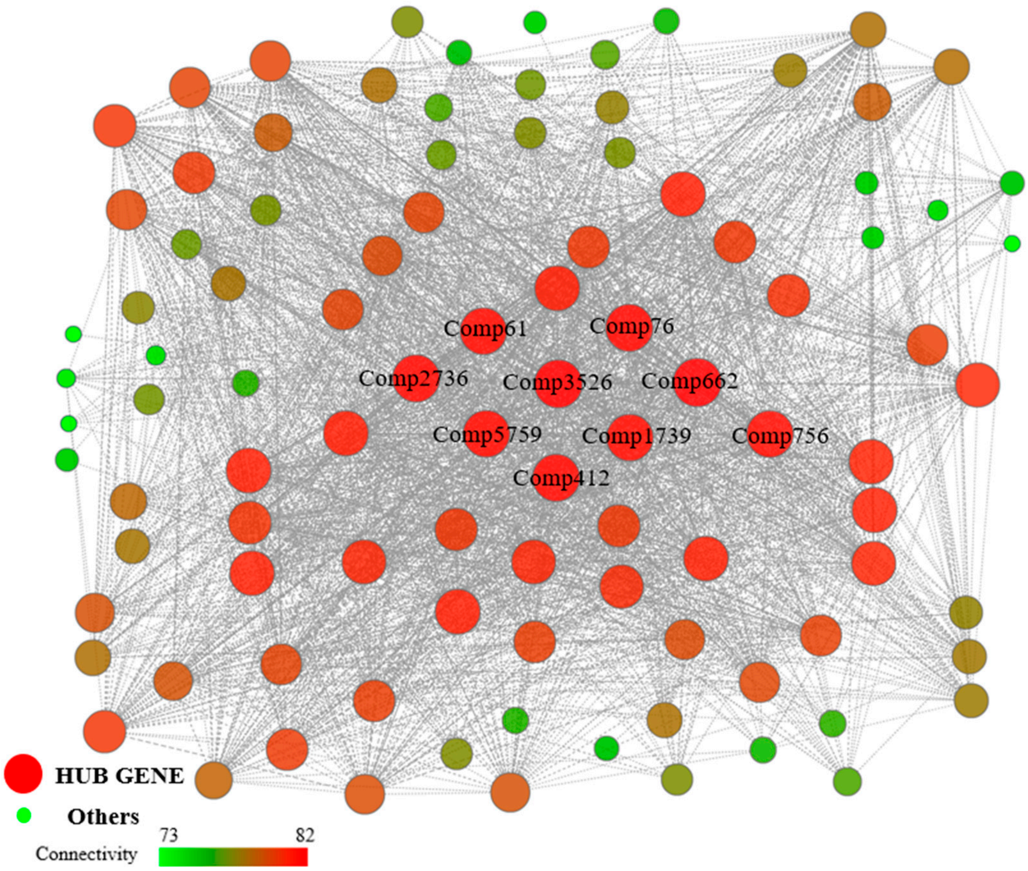

Figure S11: The correlation network of MEyelllow module.

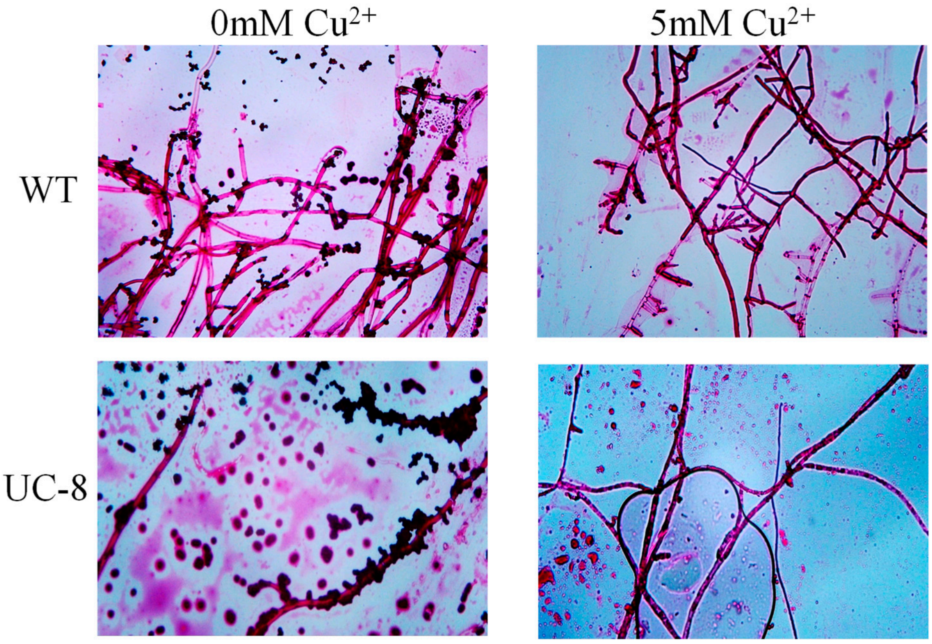

Figure S12: Microscopic morphology of hyphae (40×)

Table S1: Comparison between the results of RNA-Seq and qRT PCR

Comparison of sample：WT-TY0.5 VS W'T TY0

Unigene	Name	Definition	RNA-Seq	Q-PCR
comp1372	copA	Cu2+-exporting ATPase	2.94	-2.10
comp478	copA	Cu2+-exporting ATPase	6.50	4.29
comp1103	copA	Cu2+-exporting ATPase	3.50	6.74
comp2615	copA	Cu2+-exporting ATPase	-2.06	0.10
comp3495	PUT3	proline utilization trans-activator	0.54	3.86
comp5863	PUT3	proline utilization trans-activator	-0.83	-0.91
comp7977		proline dehydrogenase	-0.93	-2.68
comp7926		proline dehydrogenase	-1.18	-1.86
comp2884	ATP7	Cu2+-exporting ATPase	0.27	-2.84
comp3523	ATP7	Cu2+-exporting ATPase	0.86	6.26
comp13468	ATP7	Cu2+-exporting ATPase	3.11	-6.42
comp17667	ATP7	Cu2+-exporting ATPase	-2.17	-2.00
comp28678	ATP7	Cu2+-exporting ATPase	-3.08	-2.34
comp839	PUT3	proline utilization trans-activator	-2.08	0.69
comp3482	PSTPIP 1	proline-serine-threonine phosphatase interacting protein 1	0.94	4.13
comp5284	PUT3	proline utilization trans-activator	-0.33	0.65
comp5687	PUT3	proline utilization trans-activator	1.45	1.94
comp6763	PUT3	proline utilization trans-activator	-2.12	3.92
comp8290	PUT3	proline utilization trans-activator	1.28	3.41
comp1075		Cell wall integrity and stress response component 4	0.29	-0.48
comp529	PPP2C	protein phosphatase 2 (formerly 2A), catalytic subunit	0.02	0.12
comp1599	ALR	magnesium transporter	1.05	0.39
comp1895	MTR4	ATP-dependent RNA helicase DOB1	0.16	-0.85
comp706		ATP synthase H chain	0.09	-0.47
comp637		Cytochrome P450 52A12	1.53	0.06
comp978	YME1	ATP-dependent metalloprotease	0.02	0.40
comp1026	AP1F	fungal AP-1-like factor	1.03	-0.32
comp1141	K07126	Chitin synthase regulatory factor 3	-0.49	-1.18
comp1183		Transcription initiation factor IIA subunit 2	0.42	-0.22
comp1353	EIF4B	translation initiation factor 4B	0.32	-0.76
comp149		RNA polymerase I specific transcription initiation factor Rrn7	-2.16	0.61
comp1961		transcription factor btf3	-0.53	-2.42
comp658		Transcription factor, fork head, conserved site	0.58	0.54
comp665	EIF6	translation initiation factor 6	-0.02	-0.47
comp514		Histone transcription regulator 3-like protein	0.52	0.47

comp1081		Signaling mucin MSB2	1.61	0.34
comp1417	VAPA	vesicle-associated membrane protein-associated protein A	1.45	1.02
comp578		putative cell wall proline rich protein	0.94	-0.68

Table S2: Comparison between the results of RNA-Seq and qRT PCR

Comparison of sample：EC-6-TY3 VS W'T TY3

Unigene	Name	Definition	RNA-Seq	Q-PCR
comp1372	copA	Cu^{2+}-exporting ATPase	-1.38	1.37
comp478	copA	Cu2+-exporting ATPase	-1.45	1.02
comp1103	copA	Cu2+-exporting ATPase	-2.01	1.15
comp2615	copA	Cu2+-exporting ATPase	-3.75	6.32
comp3495	PUT3	proline utilization trans-activator	-2.22	0.97
comp5863	PUT3	proline utilization trans-activator	-1.38	1.62
comp7977		proline dehydrogenase	-1.55	1.40
comp7926		proline dehydrogenase	2.60	0.06
comp2884	ATP7	Cu2+-exporting ATPase	-2.99	-10.55
comp3523	ATP7	Cu2+-exporting ATPase	0.12	0.76
comp13468	ATP7	Cu2+-exporting ATPase	-2.22	7.71
comp17667	ATP7	Cu2+-exporting ATPase	0.24	0.17
comp28678	ATP7	Cu2+-exporting ATPase	-0.29	3.53
comp839	PUT3	proline utilization trans-activator	1.72	2.90
comp3482	PSTPIP1	proline-serine-threonine phosphatase interacting protein 1	2.12	1.01
comp5284	PUT3	proline utilization trans-activator	0.93	0.90
comp5687	PUT3	proline utilization trans-activator	2.60	0.87
comp6763	PUT3	proline utilization trans-activator	2.91	0.86
comp8290	PUT3	proline utilization trans-activator	-0.66	0.22
comp1075		Cell wall integrity and stress response component 4	0.20	1.87
comp529	PPP2C	protein phosphatase 2 (formerly 2A), catalytic subunit	1.52	7.19
comp1599	ALR	magnesium transporter	-1.09	1.43
comp1895	MTR4	ATP-dependent RNA helicase DOB1	0.38	0.93
comp706		ATP synthase H chain	1.63	1.84
comp637		Cytochrome P450 52A12	-1.94	2.61
comp978	YME1	ATP-dependent metalloprotease	0.37	2.96
comp1026	AP1F	fungal AP-1-like factor	0.53	4.35
comp1141	K07126	Chitin synthase regulatory factor 3	0.87	2.37
comp1183		Transcription initiation factor IIA subunit 2	-0.22	1.45
comp1353	EIF4B	translation initiation factor 4B	-0.13	2.00
comp149		RNA polymerase I specific transcription initiation factor Rrn7	-0.71	2.59
comp1961		transcription factor btf3	1.01	1.59

comp658		Transcription factor, fork head, conserved site	0.56	3.21
comp665	EIF6	translation initiation factor 6	-0.98	2.02
comp514		Histone transcription regulator 3-like protein	0.36	0.95
comp1081		Signaling mucin MSB2	2.05	0.35
comp1417	VAPA	vesicle-associated membrane protein-associated protein A	0.43	2.18
comp578		putative cell wall proline rich protein	-0.52	1.92

Table S3: Comparison between the results of RNA-Seq and qRT PCR

Comparison of sample：EC-6-TY0.5 VS EC-6 TY0

Unigene	Name	Definition	RNA-Seq	Q-PCR
comp1372	copA	Cu2+-exporting ATPase	2.82	3.67
comp478	copA	Cu2+-exporting ATPase	4.15	0.24
comp1103	copA	Cu2+-exporting ATPase	2.99	2.22
comp2615	copA	Cu2+-exporting ATPase	Inf	0.28
comp3495	PUT3	proline utilization trans-activator	0.45	-0.01
comp5863	PUT3	proline utilization trans-activator	-2.55	-0.43
comp7977		proline dehydrogenase	1.19	1.43
comp7926		proline dehydrogenase	0.92	2.86
comp2884	ATP7	Cu2+-exporting ATPase	-2.24	-2.65
comp3523	ATP7	Cu2+-exporting ATPase	-0.10	0.97
comp13468	ATP7	Cu2+-exporting ATPase	1.74	-0.16
comp17667	ATP7	Cu2+-exporting ATPase	-0.06	0.58
comp28678	ATP7	Cu2+-exporting ATPase	4.57	3.80
comp839	PUT3	proline utilization trans-activator	-3.36	2.15
comp3482	PSTPIP1	proline-serine-threonine phosphatase interacting protein 1	1.11	1.47
comp5284	PUT3	proline utilization trans-activator	1.51	0.45
comp5687	PUT3	proline utilization trans-activator	1.54	1.50
comp6763	PUT3	proline utilization trans-activator	0.40	2.90
comp8290	PUT3	proline utilization trans-activator	1.99	0.47
comp1075		Cell wall integrity and stress response component 4	0.42	2.98
comp529	PPP2C	protein phosphatase 2 (formerly 2A), catalytic subunit	-0.35	3.26
comp1599	ALR	magnesium transporter	2.00	3.20
comp1895	MTR4	ATP-dependent RNA helicase DOB1	-0.29	0.32
comp706		ATP synthase H chain	0.36	2.13
comp637		Cytochrome P450 52A12	1.58	3.07
comp978	YME1	ATP-dependent metalloprotease	0.29	0.45
comp1026	AP1F	fungal AP-1-like factor	1.22	-0.10
comp1141	K07126	Chitin synthase regulatory factor 3	-0.84	0.30
comp1183		Transcription initiation factor IIA subunit 2	-0.12	0.99
comp1353	EIF4B	translation initiation factor 4B	-0.10	-0.44

comp149		RNA polymerase I specific transcription initiation factor Rrn7	-1.61	1.10
comp1961		transcription factor btf3	-1.65	-0.60
comp658		Transcription factor, fork head, conserved site	0.12	1.76
comp665	EIF6	translation initiation factor 6	-0.98	0.46
comp514		Histone transcription regulator 3-like protein	0.15	3.15
comp1081		Signaling mucin MSB2	0.80	1.17
comp1417	VAPA	vesicle-associated membrane protein-associated protein A	0.67	1.76
comp578		putative cell wall proline rich protein	0.51	0.92

Table S4: Comparison between the results of RNA-Seq and qRT PCR

Comparison of sample：EC-6-TY3 VS EC-6 TY0

Unigene	Name	Definition	RNA-Seq	Q-PCR
comp1372	copA	Cu2+-exporting ATPase	2.36	5.04
comp478	copA	Cu2+-exporting ATPase	4.19	1.66
comp1103	copA	Cu2+-exporting ATPase	1.80	3.39
comp2615	copA	Cu2+-exporting ATPase	Inf	0.81
comp3495	PUT3	proline utilization trans-activator	-0.95	-0.61
comp5863	PUT3	proline utilization trans-activator	-6.45	-2.14
comp7977		proline dehydrogenase	-2.37	1.23
comp7926		proline dehydrogenase	2.40	-0.02
comp2884	ATP7	Cu2+-exporting ATPase	5.55	-1.29
comp3523	ATP7	Cu2+-exporting ATPase	0.31	0.29
comp13468	ATP7	Cu2+-exporting ATPase	0.00	-1.23
comp17667	ATP7	Cu2+-exporting ATPase	-0.57	-0.31
comp28678	ATP7	Cu2+-exporting ATPase	-1.29	3.62
comp839	PUT3	proline utilization trans-activator	-3.00	3.66
comp3482	PSTPIP1	proline-serine-threonine phosphatase interacting protein 1	2.22	0.19
comp5284	PUT3	proline utilization trans-activator	-0.63	-0.47
comp5687	PUT3	proline utilization trans-activator	3.17	1.12
comp6763	PUT3	proline utilization trans-activator	2.32	0.63
comp8290	PUT3	proline utilization trans-activator	-1.87	1.31
comp1075		Cell wall integrity and stress response component 4	1.20	1.28
comp529	PPP2C	protein phosphatase 2 (formerly 2A), catalytic subunit	2.42	0.51
comp1599	ALR	magnesium transporter	1.73	1.02
comp1895	MTR4	ATP-dependent RNA helicase DOB1	0.73	-0.18
comp706		ATP synthase H chain	1.98	0.21
comp637		Cytochrome P450 52A12	0.93	0.87
comp978	YME1	ATP-dependent metalloprotease	0.69	-0.27
comp1026	AP1F	fungal AP-1-like factor	1.29	-1.46

comp1141	K07126	Chitin synthase regulatory factor 3	0.41	-0.73
comp1183		Transcription initiation factor IIA subunit 2	0.38	-1.25
comp1353	EIF4B	translation initiation factor 4B	0.43	-1.29
comp149		RNA polymerase I specific transcription initiation factor Rrn7	-2.10	-1.26
comp1961		transcription factor btf3	1.73	-1.56
comp658		Transcription factor, fork head, conserved site	0.91	0.37
comp665	EIF6	translation initiation factor 6	2.04	1.00
comp514		Histone transcription regulator 3-like protein	-0.10	1.33
comp1081		Signaling mucin MSB2	1.85	-0.21
comp1417	VAPA	vesicle-associated membrane protein-associated protein A	1.74	-0.20
comp578		putative cell wall proline rich protein	0.68	-0.10

Table S5: Comparison between the results of RNA-Seq and qRT PCR

Comparison of sample：EC-6 TY0 VS WT TY0

Unigene	Name	Definition	RNA-Seq	Q-PCR
comp1372	copA	Cu2+-exporting ATPase	-0.27	-6.46
comp478	copA	Cu2+-exporting ATPase	0.32	3.88
comp1103	copA	Cu2+-exporting ATPase	0.28	2.48
comp2615	copA	Cu2+-exporting ATPase	—	-6.07
comp3495	PUT3	proline utilization trans-activator	0.35	3.74
comp5863	PUT3	proline utilization trans-activator	1.70	0.99
comp7977		proline dehydrogenase	-0.59	3.06
comp7926		proline dehydrogenase	-1.14	1.99
comp2884	ATP7	Cu2+-exporting ATPase	-8.99	-11.57
comp3523	ATP7	Cu2+-exporting ATPase	0.66	4.55
comp13468	ATP7	Cu2+-exporting ATPase	-1.42	-10.66
comp17667	ATP7	Cu2+-exporting ATPase	-0.23	0.85
comp28678	ATP7	Cu2+-exporting ATPase	0.87	-1.66
comp839	PUT3	proline utilization trans-activator	0.58	-1.52
comp3482	PSTPIP1	proline-serine-threonine phosphatase interacting protein 1	-0.05	4.76
comp5284	PUT3	proline utilization trans-activator	0.28	1.23
comp5687	PUT3	proline utilization trans-activator	0.36	2.20
comp6763	PUT3	proline utilization trans-activator	-0.69	3.34
comp8290	PUT3	proline utilization trans-activator	0.21	3.22
comp1075		Cell wall integrity and stress response component 4	0.25	-2.39
comp529	PPP2C	protein phosphatase 2 (formerly 2A), catalytic subunit	0.19	-1.38
comp1599	ALR	magnesium transporter	-0.19	-1.43
comp1895	MTR4	ATP-dependent RNA helicase DOB1	0.03	-0.54
comp706		ATP synthase H chain	0.07	-1.67

comp637		Cytochrome P450 52A12	0.40	-1.89
comp978	YME1	ATP-dependent metalloprotease	0.04	0.04
comp1026	AP1F	fungal AP-1-like factor	0.46	-0.23
comp1141	K07126	Chitin synthase regulatory factor 3	0.13	-1.03
comp1183		Transcription initiation factor IIA subunit 2	0.39	-0.02
comp1353	EIF4B	translation initiation factor 4B	0.22	-0.19
comp149		RNA polymerase I specific transcription initiation factor Rrn7	-0.15	-0.35
comp1961		transcription factor btf3	0.85	-1.34
comp658		Transcription factor, fork head, conserved site	0.13	-0.55
comp665	EIF6	translation initiation factor 6	0.61	-0.30
comp514		Histone transcription regulator 3-like protein	0.45	-2.15
comp1081		Signaling mucin MSB2	0.55	-0.38
comp1417	VAPA	vesicle-associated membrane protein-associated protein A	0.54	-1.49
comp578		putative cell wall proline rich protein	0.38	-0.91

Table S6: Comparison between the results of RNA-Seq and qRT PCR

Comparison of sample：EC-6 TY0.5 VS WT TY0.5

Unigene	Name	Definition	RNA-Seq	Q-PCR
comp1372	copA	Cu2+-exporting ATPase	-0.38	-0.68
comp478	copA	Cu2+-exporting ATPase	-2.04	-0.17
comp1103	copA	Cu2+-exporting ATPase	-0.23	-2.04
comp2615	copA	Cu2+-exporting ATPase	-8.86	-5.88
comp3495	PUT3	proline utilization trans-activator	0.27	-0.14
comp5863	PUT3	proline utilization trans-activator	-0.02	-0.51
comp7977		proline dehydrogenase	1.53	1.06
comp7926		proline dehydrogenase	0.96	2.73
comp2884	ATP7	Cu2+-exporting ATPase	-10.85	-11.38
comp3523	ATP7	Cu2+-exporting ATPase	-0.30	-0.74
comp13468	ATP7	Cu2+-exporting ATPase	-2.79	-4.40
comp17667	ATP7	Cu2+-exporting ATPase	1.87	1.73
comp28678	ATP7	Cu2+-exporting ATPase	5.47	4.48
comp839	PUT3	proline utilization trans-activator	-0.71	-0.06
comp3482	PSTPIP1	proline-serine-threonine phosphatase interacting protein 1	0.12	2.10
comp5284	PUT3	proline utilization trans-activator	2.12	1.04
comp5687	PUT3	proline utilization trans-activator	0.45	1.77
comp6763	PUT3	proline utilization trans-activator	1.83	2.32
comp8290	PUT3	proline utilization trans-activator	0.93	0.28
comp1075		Cell wall integrity and stress response component 4	0.37	1.07
comp529	PPP2C	protein phosphatase 2 (formerly 2A), catalytic subunit	-0.18	1.76

comp1599	ALR	magnesium transporter	0.75	1.39
comp1895	MTR4	ATP-dependent RNA helicase DOB1	-0.42	0.64
comp706		ATP synthase H chain	0.34	0.93
comp637		Cytochrome P450 52A12	0.44	1.13
comp978	YME1	ATP-dependent metalloprotease	0.31	0.09
comp1026	AP1F	fungal AP-1-like factor	0.64	-0.01
comp1141	K07126	Chitin synthase regulatory factor 3	-0.22	0.44
comp1183		Transcription initiation factor IIA subunit 2	-0.15	1.19
comp1353	EIF4B	translation initiation factor 4B	-0.20	0.13
comp149		RNA polymerase I specific transcription initiation factor Rrn7	0.40	0.14
comp1961		transcription factor btf3	-0.27	0.48
comp658		Transcription factor, fork head, conserved site	-0.34	0.67
comp665	EIF6	translation initiation factor 6	-0.35	0.63
comp514		Histone transcription regulator 3-like protein	0.08	0.54
comp1081		Signaling mucin MSB2	-0.27	0.45
comp1417	VAPA	vesicle-associated membrane protein-associated protein A	-0.24	-0.75
comp578		putative cell wall proline rich protein	-0.05	0.68

Table S7: Hub gene from WGCNA analysis based on transcriptome sequencing,

Comparison of sample: EC-6-TY0.5 VS EC-6-TY0.

Unigene	Name	Definition	fold.change
MEblue			
comp2564	-	Putative Squalene synthetase	-3.35
comp7419	-	Rhomboid protein	-
comp15121	-	Tryptophan synthase	-
comp3712	PHO84	MFS transporter, PHS family, inorganic phosphate transporter	-6.13
comp4588	CYP2U	cytochrome P450, family 2, subfamily U	-5.37
comp2621	-	Nucleoside diphosphate kinase	-7.17
GREEN			-
comp2678	-	Vacuolar-sorting protein snf7	-7.73
comp3586	-	MFS transporter	-7.66
comp6947	-	Zinc finger, C2H2	-1.37
comp6810	-	Putative Squalene synthetase	-3.05
comp4257	-	Pyruvate dehydrogenase E1 component subunit beta, mitochondrial	-
comp4356	-	rRNA 2'-O-methyltransferase fibrillarin	-7.34
comp3306	-	bZIP transcription factor AP-1/Yap1	-1.33
comp594	-	Transcription initiation factor IIA subunit 2	-1.20
comp5324	-	Rhomboid protein	-
comp6670	-	60S ribosomal protein L11	-2.61
comp4724	-	Xyloglucan-specific endo-beta-1,4-glucanase A	-

comp318	argJ	glutamate N-acetyltransferase / amino-acid N-acetyltransferase	-3.49
comp7481	-	Ribosomal protein S5, C-terminal	-
comp5144	E3.6.3.6	H+-transporting ATPase	-2.04
comp5154	-	Glucose-methanol-choline oxidoreductase	-
comp7748	-	Glyoxalase 3	-4.46
comp5671	betA, CHDH	choline dehydrogenase	-4.49
comp2602	-	ATP-dependent RNA helicase rok1	-7.29
comp2987	-	Cell wall integrity and stress response component 4	-7.45
comp1763	MPV17	protein Mpv17	-1.32
comp1603	-	Tryptophan synthase	-3.69
MEred			
comp3536	metC	cystathionine beta-lyase	0.10
comp2931	-	ATP synthase H chain	0.61
comp2427	K06867	Cytochrome P450 52A13	0.78
comp5465	-	Mitochondrial inner membrane i-AAA protease supercomplex subunit YME1	1.32
comp6342	ALG9	alpha-1,2-mannosyltransferase	1.28
comp3496	-	Glyoxalase 3	3.18
comp3253	-	Glucose-methanol-choline oxidoreductase	0.72
comp5033	-	NAD-binding Rossmann fold protein	1.15
comp3356	-	Nucleoside diphosphate kinase	0.48
comp1679	NUP93	nuclear pore complex protein Nup93	0.35
comp1562	EIF2S3	translation initiation factor 2 subunit 3	0.09
comp1572	CUL1, CDC53	cullin 1	0.65
comp4969	-	Cell wall integrity and stress response component 4	0.31
comp4998	HIP2, UBC1	ubiquitin-conjugating enzyme (huntingtin interacting protein 2)	0.54
comp1208	LIG1	DNA ligase 1	0.40
MEturquois			
comp3920	PFDN2	prefoldin subunit 2	0.12
comp245	-	Metallophosphoesterase	0.82
comp5233	serA, PHGDH	D-3-phosphoglycerate dehydrogenase	0.25
comp4037	-	NADH-ubiquinone oxidoreductase 40 kDa subunit, mitochondrial	1.93
comp1607	PLEKHA8	pleckstrin homology domain containing, family A (phosphoinositide binding specific) member 8	0.38
comp5710	OTU1, YOD1	ubiquitin thioesterase OTU1	0.38
comp638	TUBA	tubulin alpha	0.43
comp4047	mtaP	5'-methylthioadenosine phosphorylase	0.68
comp479	APC3, CDC27	anaphase-promoting complex subunit 3	-1.14
comp1016	-	Chitin synthase regulatory factor 3	0.76
comp3543	-	Histone transcription regulator 3-like protein	2.66
comp2955	STT3	dolichyl-diphosphooligosaccharide--protein glycosyltransferase	0.22
MEgreenyellow			

comp2486	-	NADH-ubiquinone oxidoreductase 40 kDa subunit, mitochondrial	3.37
comp1359	-	ATP-dependent RNA helicase rok1	1.47
comp5056	-	Rhomboid protein	6.12
comp3570	RPC34, POLR3F	DNA-directed RNA polymerase III subunit RPC6	2.02
comp5523	RDH13	retinol dehydrogenase 13	3.00
comp3669	-	Glucose-methanol-choline oxidoreductase	2.65
comp2683	K06911	Hexokinase	2.58
comp4916	-	pyrimidine nucleoside transport protein	2.64
comp409	STIP1	stress-induced-phosphoprotein 1	1.92
MEmidnightblue			
comp6540	CRNS1, ATPGD1	carnosine synthase	3.43
comp21	-	Rhomboid protein	6.26
comp6276	-	Cell wall integrity and stress response component 4	1.98
comp6159	PC, pyc	pyruvate carboxylase	2.34
comp2459	MAK16	protein MAK16	0.07
comp5889	-	serine peptidase, family S28	2.42
comp2228	ARO8	aromatic amino acid aminotransferase I	2.74
comp3809	rbsK, RBKS	ribokinase	5.30
MEgrey			
comp4810	-	transcription factor btf3	1.42
comp5877	E3.5.2.9	5-oxoprolinase (ATP-hydrolysing)	1.40
comp1305	GOT1	aspartate aminotransferase, cytoplasmic	1.64
comp3685	-	Glycoside hydrolase, superfamily	1.69
comp4244	-	Glucose-methanol-choline oxidoreductase	2.01
comp2064	-	stomatin family protein	1.87
MEcyan			
comp5161	-	Mitochondrial inner membrane i-AAA protease supercomplex subunit YME1	0.14
comp1549	NOP2	ribosomal RNA methyltransferase Nop2	0.18
comp1073	E3.1.1.-	esterase / lipase	1.18
comp5078	-	Glucose-methanol-choline oxidoreductase	-0.15
comp2364	-	Rhomboid protein	0.60
comp5253	-	Nucleoside diphosphate kinase	-0.73
comp3270	CYP3A	cytochrome P450, family 3, subfamily A	1.34
comp6738	gabD	succinate-semialdehyde dehydrogenase (NADP+)	4.36
MEsalmon			
comp164	-	Glucose-methanol-choline oxidoreductase	1.30
comp3072	-	ATP-dependent RNA helicase rok1	1.75
comp1312	BTS, CLN3	battenin	1.00
comp4373	-	Nucleoporin NUP85	1.25
comp4369	-	stomatin family protein	0.84

comp1720	-	Cell wall integrity and stress response component 4	2.06
comp5403	-	Mitochondrial inner membrane i-AAA protease supercomplex subunit YME1	1.18
comp606	SLC28A	pyrimidine nucleoside transport protein	1.25
MElightcyan			-
comp7981	CSNK1E	casein kinase 1, epsilon	0.26
comp6943	CSNK1E	casein kinase 1, epsilon	0.00
comp2884	ATP7, copA	Cu2+-exporting ATPase	-
comp4494	-	Protein kinase gsk3	-
comp3488	-	Beta-hexosaminidase	-
comp3088	msrB	peptide-methionine (R)-S-oxide reductase	1.17
MEpink			
comp3514	bglX	beta-glucosidase	2.90
comp5797	GBA, srfJ	glucosylceramidase	2.16
comp2341	yfaW	L-rhamnonate dehydratase	1.77
comp2943	ABCG2.SNQ2	ATP-binding cassette, subfamily G (WHITE), member 2, SNQ2	2.43
comp2452	-	Serine/threonine-specific protein phosphatase/bis(5-nucleosyl)-tetraphosphatase	2.57
comp5373	-	ATP dependent RNA helicase (Dob1)	6.56
comp2339	MDR1	MFS transporter, DHA1 family, multidrug resistance protein	1.38
comp227	-	Translin-1	1.94
MEyellow			
comp61	EF3, TEF3	elongation factor 3	0.72
comp2736	TFCP2	transcription factor CP2 and related proteins	2.73
comp76	-	Tryptophan synthase	3.30
comp662	ALKBH2	alpha-ketoglutarate-dependent dioxygenase alkB homolog 2	0.46
comp3526	DAL	MFS transporter, ACS family, allantoate permease	4.94
comp5759	-	battenin	4.62
comp1739	LMAN2, VIP36	lectin, mannose-binding 2	-0.60
comp756	NMNAT	nicotinamide mononucleotide adenylyltransferase	0.95
comp412	-	RNA polymerase I specific transcription initiation factor Rrn7	1.43

Table S8: Hub gene from WGCNA analysis based on transcriptome sequencing,

Comparison of sample: EC-6-TY3 VS EC-6-TY0.

Unigene	Name	Definition	fold.change
MEblue			
comp2564	-	Putative Squalene synthetase	-4.49
comp7419	-	Rhomboid protein	-5.05
comp15121	-	Tryptophan synthase	-
comp3712	PHO84	MFS transporter, PHS family, inorganic phosphate transporter	-5.33
comp4588	CYP2U	cytochrome P450, family 2, subfamily U	-6.48

comp2621	-	Nucleoside diphosphate kinase	-
GREEN			-
comp2678	-	Vacuolar-sorting protein snf7	-8.05
comp3586	-	MFS transporter	-9.07
comp6947	-	Zinc finger, C2H2	-1.57
comp6810	-	Putative Squalene synthetase	-2.19
comp4257	-	Pyruvate dehydrogenase E1 component subunit beta, mitochondrial	-
comp4356	-	rRNA 2'-O-methyltransferase fibrillarin	-8.66
comp3306	-	bZIP transcription factor AP-1/Yap1	-2.11
comp594	-	Transcription initiation factor IIA subunit 2	-1.08
comp5324	-	Rhomboid protein	-
comp6670	-	60S ribosomal protein L11	-3.16
comp4724	-	Xyloglucan-specific endo-beta-1,4-glucanase A	-6.12
comp318	argJ	glutamate N-acetyltransferase / amino-acid N-acetyltransferase	-3.16
comp7481	-	Ribosomal protein S5, C-terminal	0.38
comp5144	E3.6.3.6	H+-transporting ATPase	-2.19
comp5154	-	Glucose-methanol-choline oxidoreductase	-7.81
comp7748	-	Glyoxalase 3	-3.81
comp5671	betA, CHDH	choline dehydrogenase	-6.19
comp2602	-	ATP-dependent RNA helicase rok1	-7.46
comp2987	-	Cell wall integrity and stress response component 4	-8.32
comp1763	MPV17	protein Mpv17	-1.74
comp1603	-	Tryptophan synthase	-2.88
MEred			
comp3536	metC	cystathionine beta-lyase	2.78
comp2931	-	ATP synthase H chain	2.22
comp2427	K06867	Cytochrome P450 52A13	2.84
comp5465	-	Mitochondrial inner membrane i-AAA protease supercomplex subunit YME1	3.30
comp6342	ALG9	alpha-1,2-mannosyltransferase	3.07
comp3496	-	Glyoxalase 3	6.11
comp3253	-	Glucose-methanol-choline oxidoreductase	2.17
comp5033	-	NAD-binding Rossmann fold protein	3.33
comp3356	-	Nucleoside diphosphate kinase	1.77
comp1679	NUP93	nuclear pore complex protein Nup93	1.63
comp1562	EIF2S3	translation initiation factor 2 subunit 3	1.53
comp1572	CUL1, CDC53	cullin 1	2.00
comp4969	-	Cell wall integrity and stress response component 4	2.62
comp4998	HIP2, UBC1	ubiquitin-conjugating enzyme (huntingtin interacting protein 2)	2.30
comp1208	LIG1	DNA ligase 1	2.04
MEturquois			
comp3920	PFDN2	prefoldin subunit 2	1.93
comp245	-	Metallophosphoesterase	3.95

comp5233	serA, PHGDH	D-3-phosphoglycerate dehydrogenase	3.20
comp4037	-	NADH-ubiquinone oxidoreductase 40 kDa subunit, mitochondrial	5.75
comp1607	PLEKHA8	pleckstrin homology domain containing, family A (phosphoinositide binding specific) member 8	3.43
comp5710	OTU1, YOD1	ubiquitin thioesterase OTU1	2.94
comp638	TUBA	tubulin alpha	2.82
comp4047	mtaP	5'-methylthioadenosine phosphorylase	4.12
comp479	APC3, CDC27	anaphase-promoting complex subunit 3	-1.70
comp1016	-	Chitin synthase regulatory factor 3	3.71
comp3543	-	Histone transcription regulator 3-like protein	6.12
comp2955	STT3	dolichyl-diphosphooligosaccharide--protein glycosyltransferase	3.06
MEgreenyellow			
comp2486	-	NADH-ubiquinone oxidoreductase 40 kDa subunit, mitochondrial	2.66
comp1359	-	ATP-dependent RNA helicase rok1	1.12
comp5056	-	Rhomboid protein	5.31
comp3570	RPC34, POLR3F	DNA-directed RNA polymerase III subunit RPC6	1.54
comp5523	RDH13	retinol dehydrogenase 13	2.53
comp3669	-	Glucose-methanol-choline oxidoreductase	2.23
comp2683	K06911	Hexokinase	2.13
comp4916	-	pyrimidine nucleoside transport protein	2.39
comp409	STIP1	stress-induced-phosphoprotein 1	1.46
MEmidnightblue			
comp6540	CRNS1, ATPGD1	carnosine synthase	0.46
comp21	-	Rhomboid protein	3.18
comp6276	-	Cell wall integrity and stress response component 4	0.23
comp6159	PC, pyc	pyruvate carboxylase	0.72
comp2459	MAK16	protein MAK16	0.75
comp5889	-	serine peptidase, family S28	0.66
comp2228	ARO8	aromatic amino acid aminotransferase I	0.18
comp3809	rbsK, RBKS	ribokinase	2.25
MEgrey			
comp4810	-	transcription factor btf3	0.61
comp5877	E3.5.2.9	5-oxoprolinase (ATP-hydrolysing)	0.58
comp1305	GOT1	aspartate aminotransferase, cytoplasmic	0.96
comp3685	-	Glycoside hydrolase, superfamily	0.65
comp4244	-	Glucose-methanol-choline oxidoreductase	0.73
comp2064	-	stomatin family protein	0.69
MEcyan			
comp5161	-	Mitochondrial inner membrane i-AAA protease supercomplex subunit YME1	-2.93

comp1549	NOP2	ribosomal RNA methyltransferase Nop2	-1.09
comp1073	E3.1.1.-	esterase / lipase	-4.09
comp5078	-	Glucose-methanol-choline oxidoreductase	-2.05
comp2364	-	Rhomboid protein	-1.16
comp5253	-	Nucleoside diphosphate kinase	-0.24
comp3270	CYP3A	cytochrome P450, family 3, subfamily A	2.43
comp6738	gabD	succinate-semialdehyde dehydrogenase (NADP+)	1.81
MEsalmon			
comp164	-	Glucose-methanol-choline oxidoreductase	-2.91
comp3072	-	ATP-dependent RNA helicase rok1	-2.28
comp1312	BTS, CLN3	battenin	-1.13
comp4373	-	Nucleoporin NUP85	-0.84
comp4369	-	stomatin family protein	-1.00
comp1720	-	Cell wall integrity and stress response component 4	-1.90
comp5403	-	Mitochondrial inner membrane i-AAA protease supercomplex subunit YME1	-0.97
comp606	SLC28A	pyrimidine nucleoside transport protein	-0.70
MElightcyan			-
comp7981	CSNK1E	casein kinase 1, epsilon	2.26
comp6943	CSNK1E	casein kinase 1, epsilon	2.58
comp2884	ATP7, copA	Cu2+-exporting ATPase	5.55
comp4494	-	Protein kinase gsk3	-
comp3488	-	Beta-hexosaminidase	-
comp3088	msrB	peptide-methionine (R)-S-oxide reductase	2.21
MEpink			
comp3514	bglX	beta-glucosidase	0.82
comp5797	GBA, srfJ	glucosylceramidase	0.55
comp2341	yfaW	L-rhamnonate dehydratase	-0.28
comp2943	ABCG2.SNQ2	ATP-binding cassette, subfamily G (WHITE), member 2, SNQ2	0.09
comp2452	-	Serine/threonine-specific protein phosphatase/bis(5-nucleosyl)-tetraphosphatase	0.80
comp5373	-	ATP dependent RNA helicase (Dob1)	3.54
comp2339	MDR1	MFS transporter, DHA1 family, multidrug resistance protein	0.32
comp227	-	Translin-1	0.32
MEyellow			
comp61	EF3, TEF3	elongation factor 3	0.48
comp2736	TFCP2	transcription factor CP2 and related proteins	3.52
comp76	-	Tryptophan synthase	2.80
comp662	ALKBH2	alpha-ketoglutarate-dependent dioxygenase alkB homolog 2	0.55
comp3526	DAL	MFS transporter, ACS family, allantoate permease	4.17
comp5759	-	battenin	4.58
comp1739	LMAN2, VIP36	lectin, mannose-binding 2	0.59
comp756	NMNAT	nicotinamide mononucleotide adenylyltransferase	1.22

comp412	-	RNA polymerase I specific transcription initiation factor Rrn7	1.44

Table S9: Hub gene from WGCNA analysis based on transcriptome sequencing,

Comparison of sample: WT-TY0.5 VS WT-TY0.

Unigene	Name	Definition	fold.change
MEblue			
comp2564	-	Putative Squalene synthetase	-2.96
comp7419	-	Rhomboid protein	-
comp15121	-	Tryptophan synthase	-
comp3712	PHO84	MFS transporter, PHS family, inorganic phosphate transporter	-5.12
comp4588	CYP2U	cytochrome P450, family 2, subfamily U	-8.04
comp2621	-	Nucleoside diphosphate kinase	-6.63
GREEN			
comp2678	-	Vacuolar-sorting protein snf7	-
comp3586	-	MFS transporter	-
comp6947	-	Zinc finger, C2H2	-1.99
comp6810	-	Putative Squalene synthetase	-2.71
comp4257	-	Pyruvate dehydrogenase E1 component subunit beta, mitochondrial	-3.34
comp4356	-	rRNA 2'-O-methyltransferase fibrillarin	-9.11
comp3306	-	bZIP transcription factor AP-1/Yap1	-2.10
comp594	-	Transcription initiation factor IIA subunit 2	-1.40
comp5324	-	Rhomboid protein	-
comp6670	-	60S ribosomal protein L11	-4.40
comp4724	-	Xyloglucan-specific endo-beta-1,4-glucanase A	-6.40
comp318	argJ	glutamate N-acetyltransferase / amino-acid N-acetyltransferase	-3.33
comp7481	-	Ribosomal protein S5, C-terminal	-0.03
comp5144	E3.6.3.6	H+-transporting ATPase	-2.75
comp5154	-	Glucose-methanol-choline oxidoreductase	-
comp7748	-	Glyoxalase 3	-7.29
comp5671	betA, CHDH	choline dehydrogenase	-9.06
comp2602	-	ATP-dependent RNA helicase rok1	-8.84
comp2987	-	Cell wall integrity and stress response component 4	-
comp1763	MPV17	protein Mpv17	-1.94
comp1603	-	Tryptophan synthase	-4.37
MEred			
comp3536	metC	cystathionine beta-lyase	0.97
comp2931	-	ATP synthase H chain	1.14
comp2427	K06867	Cytochrome P450 52A13	2.27
comp5465	-	Mitochondrial inner membrane i-AAA protease supercomplex subunit YME1	0.76
comp6342	ALG9	alpha-1,2-mannosyltransferase	1.20
comp3496	-	Glyoxalase 3	3.67
comp3253	-	Glucose-methanol-choline oxidoreductase	0.70
comp5033	-	NAD-binding Rossmann fold protein	1.22

comp3356	-	Nucleoside diphosphate kinase	1.05
comp1679	NUP93	nuclear pore complex protein Nup93	0.80
comp1562	EIF2S3	translation initiation factor 2 subunit 3	0.58
comp1572	CUL1, CDC53	cullin 1	0.61
comp4969	-	Cell wall integrity and stress response component 4	1.81
comp4998	HIP2, UBC1	ubiquitin-conjugating enzyme (huntingtin interacting protein 2)	0.55
comp1208	LIG1	DNA ligase 1	1.33
MEturquois			
comp3920	PFDN2	prefoldin subunit 2	0.35
comp245	-	Metallophosphoesterase	0.87
comp5233	serA, PHGDH	D-3-phosphoglycerate dehydrogenase	0.57
comp4037	-	NADH-ubiquinone oxidoreductase 40 kDa subunit, mitochondrial	1.53
comp1607	PLEKHA8	pleckstrin homology domain containing, family A (phosphoinositide binding specific) member 8	0.79
comp5710	OTU1, YOD1	ubiquitin thioesterase OTU1	0.74
comp638	TUBA	tubulin alpha	0.28
comp4047	mtaP	5'-methylthioadenosine phosphorylase	1.96
comp479	APC3, CDC27	anaphase-promoting complex subunit 3	-0.40
comp1016	-	Chitin synthase regulatory factor 3	0.63
comp3543	-	Histone transcription regulator 3-like protein	5.06
comp2955	STT3	dolichyl-diphosphooligosaccharide--protein glycosyltransferase	0.74
MEgreenyellow			
comp2486	-	NADH-ubiquinone oxidoreductase 40 kDa subunit, mitochondrial	3.31
comp1359	-	ATP-dependent RNA helicase rok1	-0.25
comp5056	-	Rhomboid protein	1.20
comp3570	RPC34, POLR3F	DNA-directed RNA polymerase III subunit RPC6	1.62
comp5523	RDH13	retinol dehydrogenase 13	0.78
comp3669	-	Glucose-methanol-choline oxidoreductase	1.62
comp2683	K06911	Hexokinase	1.35
comp4916	-	pyrimidine nucleoside transport protein	1.07
comp409	STIP1	stress-induced-phosphoprotein 1	0.98
MEmidnightblue			
comp6540	CRNS1, ATPGD1	carnosine synthase	3.07
comp21	-	Rhomboid protein	6.03
comp6276	-	Cell wall integrity and stress response component 4	2.25
comp6159	PC, pyc	pyruvate carboxylase	1.98
comp2459	MAK16	protein MAK16	0.79
comp5889	-	serine peptidase, family S28	2.00
comp2228	ARO8	aromatic amino acid aminotransferase I	2.37
comp3809	rbsK, RBKS	ribokinase	3.95
MEgrey			

comp4810	-	transcription factor btf3	2.54
comp5877	E3.5.2.9	5-oxoprolinase (ATP-hydrolysing)	3.35
comp1305	GOT1	aspartate aminotransferase, cytoplasmic	2.93
comp3685	-	Glycoside hydrolase, superfamily	2.41
comp4244	-	Glucose-methanol-choline oxidoreductase	3.84
comp2064	-	stomatin family protein	3.18
MEcyan			
comp5161	-	Mitochondrial inner membrane i-AAA protease supercomplex subunit YME1	2.63
comp1549	NOP2	ribosomal RNA methyltransferase Nop2	1.93
comp1073	E3.1.1.-	esterase / lipase	3.70
comp5078	-	Glucose-methanol-choline oxidoreductase	3.51
comp2364	-	Rhomboid protein	2.71
comp5253	-	Nucleoside diphosphate kinase	2.22
comp3270	CYP3A	cytochrome P450, family 3, subfamily A	8.97
comp6738	gabD	succinate-semialdehyde dehydrogenase (NADP+)	8.15
MEsalmon			
comp164	-	Glucose-methanol-choline oxidoreductase	2.26
comp3072	-	ATP-dependent RNA helicase rok1	2.10
comp1312	BTS, CLN3	battenin	1.45
comp4373	-	Nucleoporin NUP85	1.61
comp4369	-	stomatin family protein	1.75
comp1720	-	Cell wall integrity and stress response component 4	2.53
comp5403	-	Mitochondrial inner membrane i-AAA protease supercomplex subunit YME1	1.51
comp606	SLC28A	pyrimidine nucleoside transport protein	1.48
MElightcyan			
comp7981	CSNK1E	casein kinase 1, epsilon	0.12
comp6943	CSNK1E	casein kinase 1, epsilon	0.36
comp2884	ATP7, copA	Cu2+-exporting ATPase	0.27
comp4494	-	Protein kinase gsk3	0.66
comp3488	-	Beta-hexosaminidase	0.26
comp3088	msrB	peptide-methionine (R)-S-oxide reductase	1.05
MEpink			
comp3514	bglX	beta-glucosidase	3.22
comp5797	GBA, srfJ	glucosylceramidase	2.07
comp2341	yfaW	L-rhamnonate dehydratase	2.40
comp2943	ABCG2.SNQ2	ATP-binding cassette, subfamily G (WHITE), member 2, SNQ2	3.88
comp2452	-	Serine/threonine-specific protein phosphatase/bis(5-nucleosyl)-tetraphosphatase	3.00
comp5373	-	ATP dependent RNA helicase (Dob1)	-
comp2339	MDR1	MFS transporter, DHA1 family, multidrug resistance protein	1.79
comp227	-	Translin-1	2.41
MEyellow			

comp61	EF3, TEF3	elongation factor 3	0.86
comp2736	TFCP2	transcription factor CP2 and related proteins	2.83
comp76	-	Tryptophan synthase	2.56
comp662	ALKBH2	alpha-ketoglutarate-dependent dioxygenase alkB homolog 2	0.77
comp3526	DAL	MFS transporter, ACS family, allantoate permease	3.99
comp5759	-	battenin	4.62
comp1739	LMAN2, VIP36	lectin, mannose-binding 2	-0.12
comp756	NMNAT	nicotinamide mononucleotide adenylyltransferase	0.82
comp412	-	RNA polymerase I specific transcription initiation factor Rrn7	0.77

Table S10: Hub gene from WGCNA analysis based on transcriptome sequencing, Comparison of sample: WT-TY3 VS WT-TY0.

Unigene	Name	Definition	fold.change
MEblue			
comp2564	-	Putative Squalene synthetase	-4.87
comp7419	-	Rhomboid protein	-
comp15121	-	Tryptophan synthase	-
comp3712	PHO84	MFS transporter, PHS family, inorganic phosphate transporter	-3.76
comp4588	CYP2U	cytochrome P450, family 2, subfamily U	-8.04
comp2621	-	Nucleoside diphosphate kinase	-5.85
GREEN			
comp2678	-	Vacuolar-sorting protein snf7	-8.84
comp3586	-	MFS transporter	-8.07
comp6947	-	Zinc finger, C2H2	-2.62
comp6810	-	Putative Squalene synthetase	-4.92
comp4257	-	Pyruvate dehydrogenase E1 component subunit beta, mitochondrial	-
comp4356	-	rRNA 2'-O-methyltransferase fibrillarin	-
comp3306	-	bZIP transcription factor AP-1/Yap1	-2.46
comp594	-	Transcription initiation factor IIA subunit 2	-1.40
comp5324	-	Rhomboid protein	-
comp6670	-	60S ribosomal protein L11	-7.57
comp4724	-	Xyloglucan-specific endo-beta-1,4-glucanase A	-
comp318	argJ	glutamate N-acetyltransferase / amino-acid N-acetyltransferase	-3.70
comp7481	-	Ribosomal protein S5, C-terminal	0.53
comp5144	E3.6.3.6	H+-transporting ATPase	-3.14
comp5154	-	Glucose-methanol-choline oxidoreductase	-
comp7748	-	Glyoxalase 3	-4.63
comp5671	betA, CHDH	choline dehydrogenase	-8.06
comp2602	-	ATP-dependent RNA helicase rok1	-
comp2987	-	Cell wall integrity and stress response component 4	-8.03
comp1763	MPV17	protein Mpv17	-2.43
comp1603	-	Tryptophan synthase	-4.14

MEred			
comp3536	metC	cystathionine beta-lyase	2.51
comp2931	-	ATP synthase H chain	1.75
comp2427	K06867	Cytochrome P450 52A13	3.31
comp5465	-	Mitochondrial inner membrane i-AAA protease supercomplex subunit YME1	1.90
comp6342	ALG9	alpha-1,2-mannosyltransferase	2.52
comp3496	-	Glyoxalase 3	5.40
comp3253	-	Glucose-methanol-choline oxidoreductase	1.68
comp5033	-	NAD-binding Rossmann fold protein	2.73
comp3356	-	Nucleoside diphosphate kinase	1.69
comp1679	NUP93	nuclear pore complex protein Nup93	1.48
comp1562	EIF2S3	translation initiation factor 2 subunit 3	1.27
comp1572	CUL1, CDC53	cullin 1	1.64
comp4969	-	Cell wall integrity and stress response component 4	3.18
comp4998	HIP2, UBC1	ubiquitin-conjugating enzyme (huntingtin interacting protein 2)	1.41
comp1208	LIG1	DNA ligase 1	2.16
MEturquois			
comp3920	PFDN2	prefoldin subunit 2	0.23
comp245	-	Metallophosphoesterase	0.64
comp5233	serA, PHGDH	D-3-phosphoglycerate dehydrogenase	0.41
comp4037	-	NADH-ubiquinone oxidoreductase 40 kDa subunit, mitochondrial	1.21
comp1607	PLEKHA8	pleckstrin homology domain containing, family A (phosphoinositide binding specific) member 8	1.11
comp5710	OTU1, YOD1	ubiquitin thioesterase OTU1	0.87
comp638	TUBA	tubulin alpha	0.19
comp4047	mtaP	5'-methylthioadenosine phosphorylase	2.06
comp479	APC3, CDC27	anaphase-promoting complex subunit 3	-2.24
comp1016	-	Chitin synthase regulatory factor 3	0.51
comp3543	-	Histone transcription regulator 3-like protein	5.28
comp2955	STT3	dolichyl-diphosphooligosaccharide--protein glycosyltransferase	1.01
MEgreenyellow			
comp2486	-	NADH-ubiquinone oxidoreductase 40 kDa subunit, mitochondrial	1.84
comp1359	-	ATP-dependent RNA helicase rok1	-0.02
comp5056	-	Rhomboid protein	-1.62
comp3570	RPC34, POLR3F	DNA-directed RNA polymerase III subunit RPC6	0.36
comp5523	RDH13	retinol dehydrogenase 13	-2.52
comp3669	-	Glucose-methanol-choline oxidoreductase	0.64
comp2683	K06911	Hexokinase	1.18
comp4916	-	pyrimidine nucleoside transport protein	-0.04

comp409	STIP1	stress-induced-phosphoprotein 1	-0.65
MEmidnightblue			
comp6540	CRNS1, ATPGD1	carnosine synthase	-1.28
comp21	-	Rhomboid protein	4.07
comp6276	-	Cell wall integrity and stress response component 4	1.12
comp6159	PC, pyc	pyruvate carboxylase	0.80
comp2459	MAK16	protein MAK16	1.03
comp5889	-	serine peptidase, family S28	0.05
comp2228	ARO8	aromatic amino acid aminotransferase I	0.97
comp3809	rbsK, RBKS	ribokinase	1.40
MEgrey			
comp4810	-	transcription factor btf3	1.54
comp5877	E3.5.2.9	5-oxoprolinase (ATP-hydrolysing)	0.94
comp1305	GOT1	aspartate aminotransferase, cytoplasmic	1.34
comp3685	-	Glycoside hydrolase, superfamily	0.65
comp4244	-	Glucose-methanol-choline oxidoreductase	2.69
comp2064	-	stomatin family protein	0.96
MEcyan			
comp5161	-	Mitochondrial inner membrane i-AAA protease supercomplex subunit YME1	0.32
comp1549	NOP2	ribosomal RNA methyltransferase Nop2	0.32
comp1073	E3.1.1.-	esterase / lipase	-0.39
comp5078	-	Glucose-methanol-choline oxidoreductase	1.04
comp2364	-	Rhomboid protein	-0.25
comp5253	-	Nucleoside diphosphate kinase	-0.76
comp3270	CYP3A	cytochrome P450, family 3, subfamily A	2.09
comp6738	gabD	succinate-semialdehyde dehydrogenase (NADP+)	0.00
MEsalmon			
comp164	-	Glucose-methanol-choline oxidoreductase	0.50
comp3072	-	ATP-dependent RNA helicase rok1	-1.28
comp1312	BTS, CLN3	battenin	-0.03
comp4373	-	Nucleoporin NUP85	-0.24
comp4369	-	stomatin family protein	0.26
comp1720	-	Cell wall integrity and stress response component 4	0.11
comp5403	-	Mitochondrial inner membrane i-AAA protease supercomplex subunit YME1	-0.25
comp606	SLC28A	pyrimidine nucleoside transport protein	-0.60
MElightcyan			
comp7981	CSNK1E	casein kinase 1, epsilon	-1.10
comp6943	CSNK1E	casein kinase 1, epsilon	-0.22
comp2884	ATP7, copA	Cu2+-exporting ATPase	-0.45
comp4494	-	Protein kinase gsk3	-0.05
comp3488	-	Beta-hexosaminidase	-0.26

comp3088	msrB	peptide-methionine (R)-S-oxide reductase	1.51
MEpink			
comp3514	bglX	beta-glucosidase	3.62
comp5797	GBA, srfJ	glucosylceramidase	2.17
comp2341	yfaW	L-rhamnonate dehydratase	2.52
comp2943	ABCG2.SNQ2	ATP-binding cassette, subfamily G (WHITE), member 2, SNQ2	4.11
comp2452	-	Serine/threonine-specific protein phosphatase/bis(5-nucleosyl)-tetraphosphatase	2.95
comp5373	-	ATP dependent RNA helicase (Dob1)	-
comp2339	MDR1	MFS transporter, DHA1 family, multidrug resistance protein	1.78
comp227	-	Translin-1	2.83
MEyellow			
comp61	EF3, TEF3	elongation factor 3	2.63
comp2736	TFCP2	transcription factor CP2 and related proteins	5.65
comp76	-	Tryptophan synthase	6.45
comp662	ALKBH2	alpha-ketoglutarate-dependent dioxygenase alkB homolog 2	2.35
comp3526	DAL	MFS transporter, ACS family, allantoate permease	8.05
comp5759	-	battenin	4.18
comp1739	LMAN2, VIP36	lectin, mannose-binding 2	-0.25
comp756	NMNAT	nicotinamide mononucleotide adenylyltransferase	2.62
comp412	-	RNA polymerase I specific transcription initiation factor Rrn7	3.57

Table S11: Hub gene from WGCNA analysis based on transcriptome sequencing,
Comparison of sample: EC-6-TY0 VS WT-TY0.

Unigene	Name	Definition	fold.change
MEblue			
comp2564	-	Putative Squalene synthetase	0.15
comp7419	-	Rhomboid protein	1.16
comp15121	-	Tryptophan synthase	-
comp3712	PHO84	MFS transporter, PHS family, inorganic phosphate transporter	0.96
comp4588	CYP2U	cytochrome P450, family 2, subfamily U	1.14
comp2621	-	Nucleoside diphosphate kinase	1.19
GREEN			
comp2678	-	Vacuolar-sorting protein snf7	-0.59
comp3586	-	MFS transporter	-0.42
comp6947	-	Zinc finger, C2H2	-0.57
comp6810	-	Putative Squalene synthetase	-0.59
comp4257	-	Pyruvate dehydrogenase E1 component subunit beta, mitochondrial	-0.58
comp4356	-	rRNA 2'-O-methyltransferase fibrillarin	-0.45
comp3306	-	bZIP transcription factor AP-1/Yap1	-0.38
comp594	-	Transcription initiation factor IIA subunit 2	-0.29
comp5324	-	Rhomboid protein	-0.45
comp6670	-	60S ribosomal protein L11	-0.50

comp4724	-	Xyloglucan-specific endo-beta-1,4-glucanase A	-0.63
comp318	argJ	glutamate N-acetyltransferase / amino-acid N-acetyltransferase	-0.50
comp7481	-	Ribosomal protein S5, C-terminal	0.03
comp5144	E3.6.3.6	H+-transporting ATPase	-0.38
comp5154	-	Glucose-methanol-choline oxidoreductase	-0.51
comp7748	-	Glyoxalase 3	-0.60
comp5671	betA, CHDH	choline dehydrogenase	-0.87
comp2602	-	ATP-dependent RNA helicase rok1	-0.38
comp2987	-	Cell wall integrity and stress response component 4	-0.83
comp1763	MPV17	protein Mpv17	-0.41
comp1603	-	Tryptophan synthase	-0.58
MEred			
comp3536	metC	cystathionine beta-lyase	0.31
comp2931	-	ATP synthase H chain	0.07
comp2427	K06867	Cytochrome P450 52A13	0.97
comp5465	-	Mitochondrial inner membrane i-AAA protease supercomplex subunit YME1	-0.80
comp6342	ALG9	alpha-1,2-mannosyltransferase	0.08
comp3496	-	Glyoxalase 3	-0.06
comp3253	-	Glucose-methanol-choline oxidoreductase	0.08
comp5033	-	NAD-binding Rossmann fold protein	0.07
comp3356	-	Nucleoside diphosphate kinase	0.38
comp1679	NUP93	nuclear pore complex protein Nup93	0.29
comp1562	EIF2S3	translation initiation factor 2 subunit 3	0.23
comp1572	CUL1, CDC53	cullin 1	0.13
comp4969	-	Cell wall integrity and stress response component 4	0.98
comp4998	HIP2, UBC1	ubiquitin-conjugating enzyme (huntingtin interacting protein 2)	-0.38
comp1208	LIG1	DNA ligase 1	0.54
MEturquois			
comp3920	PFDN2	prefoldin subunit 2	0.22
comp245	-	Metallophosphoesterase	0.21
comp5233	serA, PHGDH	D-3-phosphoglycerate dehydrogenase	0.05
comp4037	-	NADH-ubiquinone oxidoreductase 40 kDa subunit, mitochondrial	0.10
comp1607	PLEKHA8	pleckstrin homology domain containing, family A (phosphoinositide binding specific) member 8	0.22
comp5710	OTU1, YOD1	ubiquitin thioesterase OTU1	0.21
comp638	TUBA	tubulin alpha	-0.09
comp4047	mtaP	5'-methylthioadenosine phosphorylase	0.75
comp479	APC3, CDC27	anaphase-promoting complex subunit 3	-0.10
comp1016	-	Chitin synthase regulatory factor 3	0.14
comp3543	-	Histone transcription regulator 3-like protein	2.09
comp2955	STT3	dolichyl-diphosphooligosaccharide--protein glycosyltransferase	0.21
MEgreenyellow			

comp2486	-	NADH-ubiquinone oxidoreductase 40 kDa subunit, mitochondrial	1.47
comp1359	-	ATP-dependent RNA helicase rok1	-0.06
comp5056	-	Rhomboid protein	-1.72
comp3570	RPC34, POLR3F	DNA-directed RNA polymerase III subunit RPC6	1.35
comp5523	RDH13	retinol dehydrogenase 13	-0.22
comp3669	-	Glucose-methanol-choline oxidoreductase	0.65
comp2683	K06911	Hexokinase	0.70
comp4916	-	pyrimidine nucleoside transport protein	-0.39
comp409	STIP1	stress-induced-phosphoprotein 1	1.00
MEmidnightblue			
comp6540	CRNS1, ATPGD1	carnosine synthase	1.72
comp21	-	Rhomboid protein	1.77
comp6276	-	Cell wall integrity and stress response component 4	1.12
comp6159	PC, pyc	pyruvate carboxylase	0.58
comp2459	MAK16	protein MAK16	0.17
comp5889	-	serine peptidase, family S28	0.64
comp2228	ARO8	aromatic amino acid aminotransferase I	0.91
comp3809	rbsK, RBKS	ribokinase	-0.09
MEgrey			
comp4810	-	transcription factor btf3	0.89
comp5877	E3.5.2.9	5-oxoprolinase (ATP-hydrolysing)	1.40
comp1305	GOT1	aspartate aminotransferase, cytoplasmic	1.01
comp3685	-	Glycoside hydrolase, superfamily	0.47
comp4244	-	Glucose-methanol-choline oxidoreductase	1.83
comp2064	-	stomatin family protein	0.88
MEcyan			
comp5161	-	Mitochondrial inner membrane i-AAA protease supercomplex subunit YME1	1.15
comp1549	NOP2	ribosomal RNA methyltransferase Nop2	0.48
comp1073	E3.1.1.-	esterase / lipase	0.71
comp5078	-	Glucose-methanol-choline oxidoreductase	1.63
comp2364	-	Rhomboid protein	0.41
comp5253	-	Nucleoside diphosphate kinase	0.30
comp3270	CYP3A	cytochrome P450, family 3, subfamily A	2.55
comp6738	gabD	succinate-semialdehyde dehydrogenase (NADP+)	1.00
MEsalmon			
comp164	-	Glucose-methanol-choline oxidoreductase	1.04
comp3072	-	ATP-dependent RNA helicase rok1	0.63
comp1312	BTS, CLN3	battenin	0.66
comp4373	-	Nucleoporin NUP85	0.53
comp4369	-	stomatin family protein	0.91
comp1720	-	Cell wall integrity and stress response component 4	0.69

comp5403	-	Mitochondrial inner membrane i-AAA protease supercomplex subunit YME1	0.42
comp606	SLC28A	pyrimidine nucleoside transport protein	0.42
MElightcyan			
comp7981	CSNK1E	casein kinase 1, epsilon	-4.00
comp6943	CSNK1E	casein kinase 1, epsilon	-4.83
comp2884	ATP7, copA	Cu2+-exporting ATPase	-8.99
comp4494	-	Protein kinase gsk3	-
comp3488	-	Beta-hexosaminidase	-
comp3088	msrB	peptide-methionine (R)-S-oxide reductase	0.13
MEpink			
comp3514	bglX	beta-glucosidase	0.36
comp5797	GBA, srfJ	glucosylceramidase	0.09
comp2341	yfaW	L-rhamnonate dehydratase	0.48
comp2943	ABCG2.SNQ2	ATP-binding cassette, subfamily G (WHITE), member 2, SNQ2	1.56
comp2452	-	Serine/threonine-specific protein phosphatase/bis(5-nucleosyl)-tetraphosphatase	0.20
comp5373	-	ATP dependent RNA helicase (Dob1)	-
comp2339	MDR1	MFS transporter, DHA1 family, multidrug resistance protein	0.13
comp227	-	Translin-1	0.65
MEyellow			
comp61	EF3, TEF3	elongation factor 3	0.10
comp2736	TFCP2	transcription factor CP2 and related proteins	-0.39
comp76	-	Tryptophan synthase	0.28
comp662	ALKBH2	alpha-ketoglutarate-dependent dioxygenase alkB homolog 2	0.24
comp3526	DAL	MFS transporter, ACS family, allantoate permease	-0.68
comp5759	-	battenin	1.50
comp1739	LMAN2, VIP36	lectin, mannose-binding 2	0.10
comp756	NMNAT	nicotinamide mononucleotide adenylyltransferase	-0.08
comp412	-	RNA polymerase I specific transcription initiation factor Rrn7	-0.34

Table S12: Hub gene from WGCNA analysis based on transcriptome sequencing,
Comparison of sample: EC-6-TY0.5 VS WT-TY0.5

Unigene	Name	Definition	fold.change
MEblue			
comp2564	-	Putative Squalene synthetase	-0.24
comp7419	-	Rhomboid protein	-
comp15121	-	Tryptophan synthase	-
comp3712	PHO84	MFS transporter, PHS family, inorganic phosphate transporter	-0.04
comp4588	CYP2U	cytochrome P450, family 2, subfamily U	3.81
comp2621	-	Nucleoside diphosphate kinase	0.65
GREEN			
comp2678	-	Vacuolar-sorting protein snf7	-

comp3586	-	MFS transporter	-
comp6947	-	Zinc finger, C2H2	0.05
comp6810	-	Putative Squalene synthetase	-0.93
comp4257	-	Pyruvate dehydrogenase E1 component subunit beta, mitochondrial	-
comp4356	-	rRNA 2'-O-methyltransferase fibrillarin	1.32
comp3306	-	bZIP transcription factor AP-1/Yap1	0.39
comp594	-	Transcription initiation factor IIA subunit 2	-0.09
comp5324	-	Rhomboid protein	-
comp6670	-	60S ribosomal protein L11	1.29
comp4724	-	Xyloglucan-specific endo-beta-1,4-glucanase A	-
comp318	argJ	glutamate N-acetyltransferase / amino-acid N-acetyltransferase	-0.67
comp7481	-	Ribosomal protein S5, C-terminal	-
comp5144	E3.6.3.6	H+-transporting ATPase	0.33
comp5154	-	Glucose-methanol-choline oxidoreductase	-
comp7748	-	Glyoxalase 3	2.22
comp5671	betA, CHDH	choline dehydrogenase	3.70
comp2602	-	ATP-dependent RNA helicase rok1	1.17
comp2987	-	Cell wall integrity and stress response component 4	-
comp1763	MPV17	protein Mpv17	0.21
comp1603	-	Tryptophan synthase	0.11
MEred			
comp3536	metC	cystathionine beta-lyase	-0.55
comp2931	-	ATP synthase H chain	-0.46
comp2427	K06867	Cytochrome P450 52A13	-0.52
comp5465	-	Mitochondrial inner membrane i-AAA protease supercomplex subunit YME1	-0.25
comp6342	ALG9	alpha-1,2-mannosyltransferase	0.16
comp3496	-	Glyoxalase 3	-0.55
comp3253	-	Glucose-methanol-choline oxidoreductase	0.10
comp5033	-	NAD-binding Rossmann fold protein	0.00
comp3356	-	Nucleoside diphosphate kinase	-0.19
comp1679	NUP93	nuclear pore complex protein Nup93	-0.15
comp1562	EIF2S3	translation initiation factor 2 subunit 3	-0.27
comp1572	CUL1, CDC53	cullin 1	0.18
comp4969	-	Cell wall integrity and stress response component 4	-0.52
comp4998	HIP2, UBC1	ubiquitin-conjugating enzyme (huntingtin interacting protein 2)	-0.39
comp1208	LIG1	DNA ligase 1	-0.38
MEturquois			
comp3920	PFDN2	prefoldin subunit 2	-0.01
comp245	-	Metallophosphoesterase	0.16
comp5233	serA, PHGDH	D-3-phosphoglycerate dehydrogenase	-0.28
comp4037	-	NADH-ubiquinone oxidoreductase 40 kDa subunit, mitochondrial	0.49

comp1607	PLEKHA8	pleckstrin homology domain containing, family A (phosphoinositide binding specific) member 8	-0.19
comp5710	OTU1, YOD1	ubiquitin thioesterase OTU1	-0.16
comp638	TUBA	tubulin alpha	0.06
comp4047	mtaP	5'-methylthioadenosine phosphorylase	-0.52
comp479	APC3, CDC27	anaphase-promoting complex subunit 3	-0.83
comp1016	-	Chitin synthase regulatory factor 3	0.27
comp3543	-	Histone transcription regulator 3-like protein	-0.31
comp2955	STT3	dolichyl-diphosphooligosaccharide--protein glycosyltransferase	-0.31
MEgreenyellow			
comp2486	-	NADH-ubiquinone oxidoreductase 40 kDa subunit, mitochondrial	1.53
comp1359	-	ATP-dependent RNA helicase rok1	1.66
comp5056	-	Rhomboid protein	3.20
comp3570	RPC34, POLR3F	DNA-directed RNA polymerase III subunit RPC6	1.75
comp5523	RDH13	retinol dehydrogenase 13	2.00
comp3669	-	Glucose-methanol-choline oxidoreductase	1.69
comp2683	K06911	Hexokinase	1.92
comp4916	-	pyrimidine nucleoside transport protein	1.18
comp409	STIP1	stress-induced-phosphoprotein 1	1.94
MEmidnightblue			
comp6540	CRNS1, ATPGD1	carnosine synthase	2.07
comp21	-	Rhomboid protein	2.00
comp6276	-	Cell wall integrity and stress response component 4	0.84
comp6159	PC, pyc	pyruvate carboxylase	0.94
comp2459	MAK16	protein MAK16	-0.55
comp5889	-	serine peptidase, family S28	1.06
comp2228	ARO8	aromatic amino acid aminotransferase I	1.29
comp3809	rbsK, RBKS	ribokinase	1.26
MEgrey			
comp4810	-	transcription factor btf3	-0.23
comp5877	E3.5.2.9	5-oxoprolinase (ATP-hydrolysing)	-0.55
comp1305	GOT1	aspartate aminotransferase, cytoplasmic	-0.27
comp3685	-	Glycoside hydrolase, superfamily	-0.25
comp4244	-	Glucose-methanol-choline oxidoreductase	0.00
comp2064	-	stomatin family protein	-0.44
MEcyan			
comp5161	-	Mitochondrial inner membrane i-AAA protease supercomplex subunit YME1	-1.34
comp1549	NOP2	ribosomal RNA methyltransferase Nop2	-1.26
comp1073	E3.1.1.-	esterase / lipase	-1.81
comp5078	-	Glucose-methanol-choline oxidoreductase	-2.03
comp2364	-	Rhomboid protein	-1.70

comp5253	-	Nucleoside diphosphate kinase	-2.64
comp3270	CYP3A	cytochrome P450, family 3, subfamily A	-5.07
comp6738	gabD	succinate-semialdehyde dehydrogenase (NADP+)	-2.79
MEsalmon			
comp164	-	Glucose-methanol-choline oxidoreductase	0.07
comp3072	-	ATP-dependent RNA helicase rok1	0.29
comp1312	BTS, CLN3	battenin	0.21
comp4373	-	Nucleoporin NUP85	0.17
comp4369	-	stomatin family protein	0.00
comp1720	-	Cell wall integrity and stress response component 4	0.22
comp5403	-	Mitochondrial inner membrane i-AAA protease supercomplex subunit YME1	0.08
comp606	SLC28A	pyrimidine nucleoside transport protein	0.20
MElightcyan			
comp7981	CSNK1E	casein kinase 1, epsilon	-3.86
comp6943	CSNK1E	casein kinase 1, epsilon	-5.19
comp2884	ATP7, copA	Cu2+-exporting ATPase	-
comp4494	-	Protein kinase gsk3	-
comp3488	-	Beta-hexosaminidase	-
comp3088	msrB	peptide-methionine (R)-S-oxide reductase	0.25
MEpink			
comp3514	bglX	beta-glucosidase	0.03
comp5797	GBA, srfJ	glucosylceramidase	0.19
comp2341	yfaW	L-rhamnonate dehydratase	-0.15
comp2943	ABCG2.SNQ2	ATP-binding cassette, subfamily G (WHITE), member 2, SNQ2	0.11
comp2452	-	Serine/threonine-specific protein phosphatase/bis(5-nucleosyl)-tetraphosphatase	-0.23
comp5373	-	ATP dependent RNA helicase (Dob1)	-0.18
comp2339	MDR1	MFS transporter, DHA1 family, multidrug resistance protein	-0.28
comp227	-	Translin-1	0.18
MEyellow			
comp61	EF3, TEF3	elongation factor 3	-0.04
comp2736	TFCP2	transcription factor CP2 and related proteins	-0.50
comp76	-	Tryptophan synthase	1.01
comp662	ALKBH2	alpha-ketoglutarate-dependent dioxygenase alkB homolog 2	-0.06
comp3526	DAL	MFS transporter, ACS family, allantoate permease	0.28
comp5759	-	battenin	1.50
comp1739	LMAN2, VIP36	lectin, mannose-binding 2	-0.38
comp756	NMNAT	nicotinamide mononucleotide adenylyltransferase	0.06
comp412	-	RNA polymerase I specific transcription initiation factor Rrn7	0.32

Table S13: Hub gene from WGCNA analysis based on transcriptome sequencing, Comparison of sample: EC-6-TY3 VS WT-TY3

Unigene	Name	Definition	fold.change
MEblue			
comp2564	-	Putative Squalene synthetase	0.53
comp7419	-	Rhomboid protein	-
comp15121	-	Tryptophan synthase	-
comp3712	PHO84	MFS transporter, PHS family, inorganic phosphate transporter	-0.61
comp4588	CYP2U	cytochrome P450, family 2, subfamily U	2.70
comp2621	-	Nucleoside diphosphate kinase	-
GREEN			
comp2678	-	Vacuolar-sorting protein snf7	0.19
comp3586	-	MFS transporter	-1.42
comp6947	-	Zinc finger, C2H2	0.48
comp6810	-	Putative Squalene synthetase	2.15
comp4257	-	Pyruvate dehydrogenase E1 component subunit beta, mitochondrial	-
comp4356	-	rRNA 2'-O-methyltransferase fibrillarin	-
comp3306	-	bZIP transcription factor AP-1/Yap1	-0.03
comp594	-	Transcription initiation factor IIA subunit 2	0.03
comp5324	-	Rhomboid protein	-
comp6670	-	60S ribosomal protein L11	3.91
comp4724	-	Xyloglucan-specific endo-beta-1,4-glucanase A	-
comp318	argJ	glutamate N-acetyltransferase / amino-acid N-acetyltransferase	0.03
comp7481	-	Ribosomal protein S5, C-terminal	-0.12
comp5144	E3.6.3.6	H+-transporting ATPase	0.57
comp5154	-	Glucose-methanol-choline oxidoreductase	-
comp7748	-	Glyoxalase 3	0.21
comp5671	betA, CHDH	choline dehydrogenase	1.00
comp2602	-	ATP-dependent RNA helicase rok1	-
comp2987	-	Cell wall integrity and stress response component 4	-1.12
comp1763	MPV17	protein Mpv17	0.29
comp1603	-	Tryptophan synthase	0.68
MEred			
comp3536	metC	cystathionine beta-lyase	0.59
comp2931	-	ATP synthase H chain	0.54
comp2427	K06867	Cytochrome P450 52A13	0.50
comp5465	-	Mitochondrial inner membrane i-AAA protease supercomplex subunit YME1	0.61
comp6342	ALG9	alpha-1,2-mannosyltransferase	0.63
comp3496	-	Glyoxalase 3	0.64
comp3253	-	Glucose-methanol-choline oxidoreductase	0.57
comp5033	-	NAD-binding Rossmann fold protein	0.67
comp3356	-	Nucleoside diphosphate kinase	0.47
comp1679	NUP93	nuclear pore complex protein Nup93	0.44
comp1562	EIF2S3	translation initiation factor 2 subunit 3	0.48

comp1572	CUL1, CDC53	cullin 1	0.49
comp4969	-	Cell wall integrity and stress response component 4	0.42
comp4998	HIP2, UBC1	ubiquitin-conjugating enzyme (huntingtin interacting protein 2)	0.51
comp1208	LIG1	DNA ligase 1	0.42
MEturquois			
comp3920	PFDN2	prefoldin subunit 2	1.91
comp245	-	Metallophosphoesterase	3.53
comp5233	serA, PHGDH	D-3-phosphoglycerate dehydrogenase	2.83
comp4037	-	NADH-ubiquinone oxidoreductase 40 kDa subunit, mitochondrial	4.63
comp1607	PLEKHA8	pleckstrin homology domain containing, family A (phosphoinositide binding specific) member 8	2.54
comp5710	OTU1, YOD1	ubiquitin thioesterase OTU1	2.27
comp638	TUBA	tubulin alpha	2.55
comp4047	mtaP	5'-methylthioadenosine phosphorylase	2.80
comp479	APC3, CDC27	anaphase-promoting complex subunit 3	0.44
comp1016	-	Chitin synthase regulatory factor 3	3.34
comp3543	-	Histone transcription regulator 3-like protein	2.93
comp2955	STT3	dolichyl-diphosphooligosaccharide--protein glycosyltransferase	2.26
MEgreenyellow			
comp2486	-	NADH-ubiquinone oxidoreductase 40 kDa subunit, mitochondrial	2.29
comp1359	-	ATP-dependent RNA helicase rok1	1.08
comp5056	-	Rhomboid protein	5.21
comp3570	RPC34, POLR3F	DNA-directed RNA polymerase III subunit RPC6	2.53
comp5523	RDH13	retinol dehydrogenase 13	4.83
comp3669	-	Glucose-methanol-choline oxidoreductase	2.24
comp2683	K06911	Hexokinase	1.64
comp4916	-	pyrimidine nucleoside transport protein	2.03
comp409	STIP1	stress-induced-phosphoprotein 1	3.11
MEmidnightblue			
comp6540	CRNS1, ATPGD1	carnosine synthase	3.46
comp21	-	Rhomboid protein	0.88
comp6276	-	Cell wall integrity and stress response component 4	0.23
comp6159	PC, pyc	pyruvate carboxylase	0.51
comp2459	MAK16	protein MAK16	-0.11
comp5889	-	serine peptidase, family S28	1.25
comp2228	ARO8	aromatic amino acid aminotransferase I	0.12
comp3809	rbsK, RBKS	ribokinase	0.76
MEgrey			
comp4810	-	transcription factor btf3	-0.05
comp5877	E3.5.2.9	5-oxoprolinase (ATP-hydrolysing)	1.04
comp1305	GOT1	aspartate aminotransferase, cytoplasmic	0.63

comp3685	-	Glycoside hydrolase, superfamily	0.46
comp4244	-	Glucose-methanol-choline oxidoreductase	-0.13
comp2064	-	stomatin family protein	0.61
MEcyan			
comp5161	-	Mitochondrial inner membrane i-AAA protease supercomplex subunit YME1	-2.10
comp1549	NOP2	ribosomal RNA methyltransferase Nop2	-0.93
comp1073	E3.1.1.-	esterase / lipase	-2.98
comp5078	-	Glucose-methanol-choline oxidoreductase	-1.46
comp2364	-	Rhomboid protein	-0.50
comp5253	-	Nucleoside diphosphate kinase	0.83
comp3270	CYP3A	cytochrome P450, family 3, subfamily A	2.90
comp6738	gabD	succinate-semialdehyde dehydrogenase (NADP+)	2.81
MEsalmon			
comp164	-	Glucose-methanol-choline oxidoreductase	-2.37
comp3072	-	ATP-dependent RNA helicase rok1	-0.36
comp1312	BTS, CLN3	battenin	-0.44
comp4373	-	Nucleoporin NUP85	-0.08
comp4369	-	stomatin family protein	-0.35
comp1720	-	Cell wall integrity and stress response component 4	-1.32
comp5403	-	Mitochondrial inner membrane i-AAA protease supercomplex subunit YME1	-0.30
comp606	SLC28A	pyrimidine nucleoside transport protein	0.32
MElightcyan			
comp7981	CSNK1E	casein kinase 1, epsilon	-0.64
comp6943	CSNK1E	casein kinase 1, epsilon	-2.03
comp2884	ATP7, copA	Cu2+-exporting ATPase	-2.99
comp4494	-	Protein kinase gsk3	-5.45
comp3488	-	Beta-hexosaminidase	-1.79
comp3088	msrB	peptide-methionine (R)-S-oxide reductase	0.84
MEpink			
comp3514	bglX	beta-glucosidase	-2.44
comp5797	GBA, srfJ	glucosylceramidase	-1.52
comp2341	yfaW	L-rhamnonate dehydratase	-2.33
comp2943	ABCG2.SNQ2	ATP-binding cassette, subfamily G (WHITE), member 2, SNQ2	-2.46
comp2452	-	Serine/threonine-specific protein phosphatase/bis(5-nucleosyl)-tetraphosphatase	-1.95
comp5373	-	ATP dependent RNA helicase (Dob1)	-3.19
comp2339	MDR1	MFS transporter, DHA1 family, multidrug resistance protein	-1.33
comp227	-	Translin-1	-1.85
MEyellow			
comp61	EF3, TEF3	elongation factor 3	-2.05
comp2736	TFCP2	transcription factor CP2 and related proteins	-2.52
comp76	-	Tryptophan synthase	-3.38

comp662	ALKBH2	alpha-ketoglutarate-dependent dioxygenase alkB homolog 2	-1.56
comp3526	DAL	MFS transporter, ACS family, allantoate permease	-4.56
comp5759	-	battenin	1.90
comp1739	LMAN2, VIP36	lectin, mannose-binding 2	0.93
comp756	NMNAT	nicotinamide mononucleotide adenylyltransferase	-1.48
comp412	-	RNA polymerase I specific transcription initiation factor Rrn7	-2.47

Table S14: Primers used in this study

**Gene**	**Primer name**	**Sequence(5'to3')**
b-actin	b-actin 3F	ACGTTGTCCCCATCTACGAA
	b-actin 3R	GCTCAGCCAGGATCTTCATC
comp1372	1372 F	GCAGCCGAGTTCGCATAAG
	1372 R	GTTCCGCCGTATCAAGTTCAA
comp1478	478 F	GCTCGGTCGCACATACAAG
	478 R	TCACGGTTCATTCGCTGGAT
comp11103	1103 F	TTGTTGTTGACGGAGGACTTG
	1103 R	GCTCTGTAAGGCGGTGGTT
comp12884	2884 F	GCCGCATGAGAACCACATC
	2884 R	CGCTGACAAGAAGGACTACATT
comp13523	3523 F	CTGTCTAGCGTGGCGTCTT
	3523 R	GGTCTCCGTCAGTCTCATCAT
comp12615	2615 F	AGTGGACTGGATTGCGTTGT
	2615 R	CGATTGGATGGCTGCTGTTC
comp113468	13468 F	GAACCAAGGCACGAACGAT
	13468 R	AGACTTCCGACTGGCATCC
comp117667	17667 F	GTCGCCTTGATTATGGTTGGT
	17667 R	GTGTTGCCGTATTGAAGAAGTC
comp128678	28678 F	AACGGCTCAGTCGAACAGT
	28678 R	AAGTTGACGAGTCTATGCTCAC
comp1839	839 F	TCAGTGCCGTGCTCTATGG
	839 R	CGTTATCGCCGTGATATTGACT
comp13495	3495 F	CTAGGCTGTGATGCGAATGAC
	3495 R	ATGGCTTGAAGAGGTGAGGAG
comp13482	3482 F	GGCTACGAGTGGAAGATTGAC
	3482 R	TGCTCCTATCTCATCTCACAAGT

comp15284	5284 F	AGAGGAACTAGGAGGACCAGAG
	5284 R	CGTGTAGTGTGAGGTTGATGTG
comp1	5687 F	CTACCTCAACTGCCGTCTCTT
	5687 R	CGAATTGCCGTGGATGGATG
comp15863	5863 F	GACGAGGCACAAGTGTAACC
	5863 R	GGACAGTGGCGTGAATCATC
comp16763	6763 F	CGATGCGTCTGACCAACTG
	6763 R	TTCACCTCTGAGTAGCCTTCC
comp17977	7977 F	TGGCGAGACAGAAGACGATT
	7977 R	ATTGCGGATACGATGTTGTGT
comp17926	7926 F	CCATCGGTGACTTCGGAACA
	7926 R	CTTCGGTGCGTTGCTTGTG
comp18290	8290 F	AAGCGAAGCATTACAGGTTCC
	8290 R	AGGCAAGGACAAGTTACTGGAT
comp149	149F	CGTCACTGTCACTGAGCATAGGATTG
	149R	ATTCCATCGGCACTGTCTTCATCTG
comp1353	1353F	GAAGCCACTGCCGCCGATG
	1353R	GTCTGCCTGTCCTCCACAATGC
comp1081	1081F	CCTTGGATGTGTTAGCCTTGGTAGTC
	1081R	GAGCCAGCGAAGCAGGTCATTG
comp665	665F	CATCTGCCACGCCACCATCG
	665R	CAACCGCTCCTCTATACGCTGAATC
comp658	658F	TAGGTTGCTGCGTGTGCTGTTG
	658R	GGTCCGAAGAAGTCCAAGCGAAG
comp1961	1961F	CACTATACGGCGAGACAGCACAAG
	1961R	TGAGAATGGTGACACGAATAGAGTTGG
comp1353	1353F	GAAGCCACTGCCGCCGATG
	1353R	GTCTGCCTGTCCTCCACAATGC
comp1183	1183F	GACCAGTGTGCTGTTCGGAGATC
	1183R	CGTCCAGACTTCGTCGCAGAAC
comp1141	1141F	CTTGTTGACGGCAGAGCGAGAG
	1141R	GGAAGATGCGAAGCGGTGGTAC
comp1026	1026F	CCTCCTCCTCCTCATCATAGTCCATAC
	1026R	TGTGGCTCGGCAGGCTGTC

comp514	514F	CATCAGGCAACAAGGCGGAGAG
	514R	ATATGTGGAAGAGTGAGCAAGCAAGG
comp1625	1625F	GCATCCTCACCACGCCAATCG
	1625R	CGGTGCGAGTGATAGCGACATC
comp978	978F	ACCAACTACGACAGCCTCTCCTC
	978R	AGCACCTTCTCCATCTCCTCCTTG
comp73	73F	CGCACATACCGCCAGAAGAAGG
	73R	CAGAGTTGAGGAGATCCGACACATTC
comp637	637F	TCCGTCCGAGAACAATACTACAAGAAC
	637R	ATGAGCGAAGACAAGCCAAGAAGG
comp882	882F	TCGTGCTGGTGCTGAGGAGAC
	882R	TTGATAACGCTCTGGTCCGAATGC
comp823	823F	AGACCAGACTGCGGACTAAGGAC
	823R	CGATGACGAGGAAGATGACGACAAG
comp706	706F	TGGTCGAATGATGCGTACTACAAGAAG
	706R	CAGTGTGGCGATTGAGGAACCG
comp659	659F	CGGATGTCGTTCAGGATCGGTTG
	659R	TGGATGAAGGCGAAGGAGGAGAG
comp464	464F	GTCATAACAAGGCAGGTAGCAGTAGG
	464R	GAACCAACGAGTCATCTACACGAGTC
comp1895	1895F	GCTTCATCGGCACGGCAGAG
	1865R	GCATCGCTCGCTTACTCCATCC
comp1852	1852F	AAGATTGAGCGTGTCGGCAAGG
	1852R	TCTTCTTGAGCGATGCGATGATGG
comp1351	1351F	TGTGTTCAAGAATCCATCCAAGTCTCC
	1351R	CCGCAAGTCTCCTACCAGCAATG
comp1074	1074F	GTCGCTGGTGTTGGTGTCTTCC
	1074R	GACGGCACGGCGGTTGTTAG
comp1664	1664F	GGTCTCCGTTCTTGATAATGCTCTCC
	1664R	ACTATTCCGAGGCTTGCTGTTGC
comp1599	1599F	TCCGAAGCCAGATTCCTCCTCAG
	1599R	ACCACAAGATAATGAAGGCAGCAGAG
comp1547	1547F	GCACCACCAGCCATCGTTCC
	1547R	ATTGCCAGTCAGACAGTGAAGAAGG

comp1705	1705F	CTGTCTCTGTTCTCCGCTCTAATCG
	1705R	GTGGCATCTCGCTTGACCTTGG
comp463	463F	CTGCTCCTCTTCGTGCTGAATCTG
	463R	GCCTTGAACGCCACCGATACAG
comp529	529F	ACCTCCACCTCCACCGTCATTC
comp529	529R	TTGTAGTTGCTGCCGCCGAAG
comp245	245F	TGCGTCACGAGTTGCCATTCAG
	245R	CCTCAGTGCGGACTTGCCTTG
comp1498	1498F	GCTGGACCTTGAGGAGATTGATTGG
	1498R	CATTCACGCCGCAACTCAACAAC
comp896	896F	AGGTGGCGATCCGAGACGATG
	896R	CTTGCTGCTGTGGTGAGAAGGTAG
comp1250	1250F	GTTACGGCAGTACCAGCAAGGAC
	1250R	ATGTTACAAGTTGGCAGGCTATCAGG
comp1075	1075F	ACTCCACCGCAACGCATAAGC
	1075R	CAGCCACAGACACAGACACAGAAG
comp578	578F	GTAGCAGGACTGTTCGTTTC
	578R	TTAAGGTCAAGACCACTGCT
comp1417	1417F	CGGATGTCGTTCAGGATCGGTTG
	1417R	TGGATGAAGGCGAAGGAGGAGAG
